# Efficacy of zinc oxide and copper oxide nanoparticles on virulence genes of avian pathogenic *E. coli (*APEC) in broilers

**DOI:** 10.1186/s12917-023-03643-y

**Published:** 2023-08-04

**Authors:** Fawzia A. El-Shenawy, Eman M. El. El-Sherbeny, Samr Kassem

**Affiliations:** 1 Bacteriology unit, Tanta lab. (AHRI), Animal Health Research Institute, Agricultural Research Center (ARC), Giza, Egypt; 2 Pharmacology unit, Tanta lab. (AHRI), Animal Health Research Institute, Agricultural Research Center (ARC), Giza, Egypt; 3 Nanomaterials research and Synthesis unit, Animal Health Research Institute (AHRI), Agricultural Research Center (ARC), Giza, Egypt

**Keywords:** Arabic gum, Zinc oxide nanoparticles, Copper oxide nanoparticles, Avian pathogenic *E. coli*, Virulence genes, Antimicrobial alternatives, Cu residues

## Abstract

**Background:**

Colibacillosis is one of the broilers’ most dominant bacterial diseases, either as a primary or a secondary infection. As *E. coli* antimicrobial drug resistance is rising; there is a need to develop new approaches to its control. In light of this, a comparative study of the *in-vitro* antibacterial activity of Arabic gum stabilized zinc and copper nanoparticles (AG-ZnNPs and AG-CuNPs) against PCR-identified field avian pathogenic *E. coli* (APEC) strains and virulence genes (*ibeA, hlyA, iss, pap C* and *ompA*) was applied to study the therapeutic effect of zinc and copper nanoparticles to be used as an antibiotic alternative (Nanobiotic). Furthermore, the *in-vivo* effects of CuNPs were evaluated. Additionally, the CuNPs liver and muscle residues with or without infection were examined. The eighty broilers were divided into four groups; G1: negative control, G2: infected control with *E. coli* O17, G3: non-infected treated (AG-CuNPs 50 mg/kg body weight), and G4: infected treated (AG-CuNPs 50 mg/kg body weight). AG-CuNPs treatment was given to broilers for five days in drinking water.

**Results:**

*E. coli* was isolated from diseased broilers at an average incidence rate of 20% from intestinal and liver samples. All identified serotypes (O17, O78, O91, O121, and O159) were resistant to AG-ZnNPs and sensitive to AG-CuNPs. AG-CuNPs minimal inhibitory and bactericidal concentrations (MIC and MBC) for O17 were 7.5 and 60 mg/ml, respectively. Conventional uniplex PCR results showed that strain O17 contained virulence genes (*ibeA, hlyA, iss*, and *papC*), where AG-CuNPs significantly reduced the expression of all target genes when examined by Real-time quantitative PCR. Additionally, the bactericidal activity of AG-CuNPs on O17 was 100% at 20 minutes and 40 mg/ml and confirmed by transmission electron microscopy. Furthermore, no mortality was recorded in treated groups compared to G2. Subsequently, no *E. coli was* re-isolated from the liver in the G4 after treatment. The total protein, albumin, globulin, and lysozyme activity were significantly increased in G4 compared to G2, while the activities of liver enzymes (alanine aminotransferase (ALT), Gamma-glutamyl transferase (GGT), and alkaline phosphatase (ALP)) were markedly decreased in G4 compared to G2. Additionally, uric acid, creatinine, and C-reactive protein levels were decreased in G4 compared to G2. However, the liver enzymes, kidney functions, C-reactive protein levels, and Cu residues were non-significantly changed in G4 compared to G1.

**Conclusion:**

Green synthesized AG-CuNPs are recommended as an effective antimicrobial alternative against APEC strains.

**Supplementary Information:**

The online version contains supplementary material available at 10.1186/s12917-023-03643-y.

## Background

Colibacillosis is the most dominant bacterial disease in broilers. It is considered as one of the most important multi-systemic diseases. It causes various symptoms at all ages, either as a primary or secondary infection. *E. coli* that cause disease within the avian host is categorized as avian pathogenic *E. coli* (APEC). It induces localized and systemic infections, leading to poultry production loss and quick mortality [1]. Furthermore, APEC can induce human diseases using *in-vitro* and *in-vivo* models, suggesting its zoonotic potential; therefore, it is considered a public health concern [2]. Although no distinct virulence feature has been established to recognize an APEC, there are some known virulence markers or mechanisms for APEC to cause colibacillosis in poultry. The Identified virulence markers of APEC include adhesions, toxins, iron acquisition mechanisms, invasions, and plasmids [3]. Various studies have revealed that these virulence markers are rarely present in the same isolate strain; however, single or multiple virulence markers can be detected in clinical isolates with different percentages [4].

By increasing the restriction of antibiotic growth promoter usage in poultry production, it can be speculated that colibacillosis would become an even more significant problem on commercial farms. Furthermore, treating bacterial *E. coli* infections is gradually complicated by the ability of bacteria to induce resistance to antimicrobial agents. Therefore, antimicrobial drug resistance of *E. coli* is considered a crucial public health problem and attracts poultry veterinarians’ concern [5].Hence, several new approaches have been applied to control microbial infection, such as metal oxide nanoparticles (MONPs), a new class of materials for potential use in scientific research and health-related applications. Indeed, nanotechnology can be used in the pharmaceutical industry, animal health, veterinary medicine, and various extents for animal production. It is considered an effective, eco-friendly, and low-cost strategy for disease control.

Nanoparticles (NPs) have been demonstrated as proficient therapeutic agents due to their outstanding physicochemical properties, characteristics, and universally applicable physical mode of action [6].Thus, they are considered safe and effective biocidal compounds to counteract poultry bacterial infections [7]. Recently, metal nanomaterials have been used as an antimicrobial against communal infectious agents without inducing antibiotic resistance in the organisms [8].

Diverse simultaneous mechanisms of action of NPs against bacteria would make it hard for the microbes to develop resistance; thus, multiple simultaneous gene mutations inside the bacterial cell should be required to develop this resistance [9]. Additionally, some inorganic NPs are non-toxic because they contain minerals essential to the body. Most metallic NPs and metal oxide NPs have antibacterial activity, such as silver, titanium, copper oxide, and zinc oxide. Most of the antimicrobial action of NPs occurs due to their penetration and diffusion through the cell membrane of organisms, leading to the generation of oxidative stress, which destroys the microbial cells [10].

Indeed, MONPs were manufactured on a large scale and grew exponentially worldwide; therefore, they are extensively used in various fields of biomedical applications due to their increased bioavailability and endothelial cell adsorption, simple preparation processes, easy engineering to the desired size, shape, and porosity, and easy incorporation into hydrophobic and hydrophilic systems. In addition, they are less toxic than salts of the same metals, with a prolonged effect on biological objects and many novel properties compared with bulk materials [11]. Thus, easy, cheap, and eco-friendly approaches paid more attention to formulating metallic NPs to avoid the hazards of the chemical synthesis of NPs and their byproducts [12]. In light of this, Arabic gum was used in this study as a green route approach for NPs formulation. Furthermore, CuNPs gained extensive research interest due to their immense biological benefits. They exhibited beneficial effects on immunological parameters and had high bioavailability, improved growth performance, and decreased pathogenic load to enhance broilers’ health [13]. In addition, their antimicrobial activity against various gram-positive or negative bacteria was reported [14]. Moreover, ZnNPs enter the intestinal cells through direct penetration and grant more beneficial effects at low doses for both livestock and the environment, besides being highly bioavailable, exerting a superior efficacy, and being more bioactive than zinc in bulk formulation [15].They have many applications as antimicrobial abilities against some pathogens.

Different biological responses to NPs occur inside the *in-vivo* systems. Therefore, more *in-vivo* studies are needed to examine the main relationship between the used *in-vitro* dose, physicochemical features of NPs, and their mode of action in disease pathophysiology to achieve the safest effective dose for treatment, optimize therapeutic benefits, and safeguard their commercial application in the poultry industry. Furthermore, these items will be beneficial to overcome biological barriers, for instance, improving the targeting and reducing the accumulation in non-targeted cells, tissue, and organs [13].

However, several studies showed the beneficial and hazardous effects of Cu or Zn NPs as nutritional supplements, few studies reported the therapeutic effect against poultry pathogens as an antibiotic alternative (Nanobiotic). In this concern, this study aimed to explore the *in-vitro* antibacterial activities of AG-Cu and Zn NPs against various field-isolated APEC strains. Additionally, sensitivity tests, such as minimal inhibitory and bactericidal concentrations (MIC and MBC), were performed to investigate their efficacy besides using antibiotics for judging susceptibility patterns. Moreover, the strain with the highest number of virulence genes (*E. coli* O17) was used to examine the efficacy of the used CuNPs on these genes using real-time quantitative PCR (RTQ-PCR), besides estimating the effect of concentration and time on its antibacterial performance.

Furthermore, a transmission electron microscope (TEM) was used to confirm AG-CuNPs bactericidal mechanism. Subsequently, AG-CuNPs were the most effective ones. They were used *in-vivo* on broiler chickens to develop a method for predicting the therapeutic effectiveness of green synthesized nanoscale preparations as an eco-friendly approach for the biogenic production of Arabic gum-stabilized CuNPs. Moreover, their effects on clinical signs, *E. coli* re-isolation and count, non-specific immunity (lysozyme activity, total proteins, and globulin), anti-inflammatory C-reactive protein, liver enzymes activities (alanine aminotransferase (ALT), gamma-glutamyl transferase (GGT), and alkaline phosphatase (ALP)), uric acid, and creatinine levels were studied in non-infected and infected broilers. Additionally, Cu residues in both muscles and liver with/without infection were evaluated using atomic absorption spectrophotometer to avoid consumer residue problems.

## Materials and methods

### Collection of samples

Samples from the liver and intestine were obtained from diseased broiler chickens; all samples were collected under aseptic conditions and transferred to the bacteriological lab.

### Isolation and identification of E. coli from collected samples

All collected samples were cultured on nutrient broth and incubated at 37 °C/24 h for enrichment. Then, a loop full of nutrient broth was cultivated on eosin methylene blue (EMB) agar media and incubated aerobically for 24 h. Next, pure suspected colonies were examined microscopically by gram staining and tested biochemically for identification and confirmation [16]. Finally, isolated pure colonies were sub cultured on sheep blood agar plates and incubated for 24 h aerobically to detect the suspected pathogenic strain [17].

### Serological typing and identification of E. coli isolates

Obtained *E. coli* isolates were serologically identified [18] using rapid diagnostic *E. coli* antisera sets (DENKA SEIKEN Co., Japan) to diagnose Enteropathogenic types.

### Molecular detection of virulence genes in isolated APEC strains using conventional uniplex PCR (cPCR)

Procedures were performed according to the instruction of the Laboratory for Poultry Production of Zagazig lab, Animal Health Research Institute, Egypt [19]. Five serotypes of isolated samples were selected to detect the virulence genes (*ibeA, hlyA, iss, papC, and ompA*). First, the extraction of DNA was performed according to QIAamp DNA mini kit instructions. Then, PCR Master Mix was prepared according to EmeraldAmp GT PCR master mix (Takara) Code No. RR310A kit as follows, 12.5 µl of EmeraldAmp GT PCR master mix (2x premix), 4.5 µl of PCR grade water, 1 µl of forward primer (20 pmol),1 µl of reverse primer (20 pmol), and 6 µl of template DNA. Subsequently, 25 µl of this mixture was used for agarose gel electrophoresis [20]. Oligonucleotide primer sequences used in cPCR are shown in Table 1.

**Table 1:** Primers sequences, target genes, amplicon sizes and cycling conditions for conventional PCR and real time quantitative-PCR

Target gene	Primers sequences	Reverse transcription	Primary denaturation	Amplification (40 cycles)			Reference
				Secondary denaturation	Annealing (Optics on)	Extension	
**ibeA**	TGGAACCCGCTCGTAATATAC	65˚C/ 60 min.	95˚C/ 15 min.	95˚C/ 15 s.	60˚C/ 1 min.	72˚C/ 30 s.	[19]
	CTGCCTGTTCAAGCATTGCA						
**hlyA**	AACAAGGATAAGCACTGTTCTGGC						[21]
	ACCATATAAGCGGTCATTCCCGTCA						
**Iss**	ATCACATAGGATTCTGCCG						[22]
	CAGCGGAGTATAGATGCCA						
**papC**	GTGGCAGTATGAGTAATGACCGTTA						[23]
	ATATCCTTTCTGAGGGATGCAATA						
**ompA**	GGTGTTGCCAGTAACCGG						[19]
	GGTGGTGCACTGGAG TGG						
**16 S rRNA**	GACCTCGGTTTAGTTCACAGA						[24]
	CACACGCTGACGCTGACCA						

### Preparation of experimental infective dose of APEC O17strain culture for challenge assay

*E. coli* O17 serotype strain was previously isolated from liver samples and *enclosed* four virulence genes when subjected to molecular PCR. The pure *E. coli* O17 isolate was cultured on EMB and incubated aerobically at 37 °C/24 h. Colonies were picked up and inoculated on saline to obtain the stock inoculum for infection. The infective dose was enumerated to achieve 1 × 10^8^ CFU/ml, and chickens were inoculated orally at two weeks old [25].

### Preparation of metal nanoparticles

Arabic gum (Hashab) from the Acacia tree in Sudan was purchased from a local market. Zinc sulfate heptahydrate (ZnSO_4_.7H_2_O), sodium hydroxide (Na OH), copper (II) sulfate pentahydrate (CuSO_4_·5H_2_O), and L-ascorbic acid were purchased from Sigma Aldrich, USA. All the chemicals were of analytical grade and used without further purification. All the synthesis was carried out using deionized water.

#### Synthesis of arabic gum stabilized ZnNPs (AG-Zn NPs)

The ZnNPs were prepared by a green method using ZnSO_4_.7H_2_O and Na OH as precursors at a molar ratio of 1:2 and AG as stabilizing agent, according to Geetha et al. [26] with a modification. First, a concentration of 1.5% AG (0.043 g) was prepared in 100 ml of deionized water. Then 2.88 g of ZnSO_4_.7H_2_O was added to get a 0.1 M solution. The contents were kept under constant stirring using a magnetic stirrer to dissolve the zinc sulfate completely. Next, Na OH (0.2 M, 0.8 g) was prepared in 100 ml deionized water by adding dropwise and stirring for 2 h, and pH was adjusted to 10. The solution was incubated overnight and then centrifuged at 10,000 rpm at 4 °C/30 mins. The supernatant was discarded. Thus, nanoparticles were washed three times with deionized water to remove the impurities. Finally, the nanoparticles were dried at 80 °C overnight in a hot air oven for the complete conversion of zinc hydroxide into ZnNPs. A clear white color powder obtained was stored at room temperature for further use.

#### Synthesis of arabic gum stabilized CuNPs (AG-CuNPs)

The CuNPs were prepared using CuSO_4_·5H_2_O as a precursor and L-ascorbic acid as a reducing agent at a molar ratio of 1:2 according to Chawla et al. [27] with a modification. An aqueous solution of L-ascorbic acid (0.2 M, 3.52 g, 100 mL) was dropwise added to a hot aqueous solution of CuSO_4_·5H_2_O (80 °C, 0.1 M, 2.49 g, 100 mL) with AG (1.0%, 0.025 g) as capping and stabilized agent under continuous and gentle stirring using a magnetic stirrer for 2 h, pH is adjusted to 10 using Na OH (1 M). The mixture was centrifuged at 10,000 rpm at 4 °C/30 min, and pellets were collected and dried overnight in a hot air oven at 60 °C to form CuNPs. A brick red color powder obtained was stored at room temperature for further use.

### Characterization methods

The chemical composition and interaction between functional groups were detected using Fourier transform infrared spectroscopy (FT-IR), Perkin Elmer, the UK, at the scanning range of 4000 − 400 cm^− 1^. Phase identification and crystalline structure of nano powders were analyzed by using X-ray Diffractometer, XRD (SHIMADZU, XRD-6000), with Cu Ka radiation. The particle size and surface charge of synthesized NPs were characterized using a NANOTRAC-WAVE II Zetasizer (MICROTRAC, USA). After that, the morphological investigation of synthesized NPs was detected by transmission electron microscopy (TEM) (JEM-2100, JEOL).

### Cytotoxicity assay

Oral epithelial cells (OEC) were incubated in DMEM media supplemented with streptomycin, penicillin, and 10% fetal bovine serum in a 5% (v/v) humidified CO_2_ atmosphere at 37 °C. Cell viability was assessed by the sulforhodamine B (SRB) assay [28]. Briefly, aliquots of 100 µL cell suspension (5 × 10^3^ cells) were added to 96-well plates and incubated for 24 h with complete media. Next, cells were treated with another aliquot of 100 µL media containing AG-Cu and Zn NPs at various concentrations (0.31, 0.62, 1.25, 2.5, 5, 10, 20, 30, 40, and 50 mg/ml). After 72 h, cells were fixed by replacing media with 150 µL of 10% trichloroacetic acid (TCA) and incubated at 4 °C for 1 h. After washing, aliquots of 70 µL SRB solution (0.4% w/v) were added and incubated at room temperature in a dark place for 10 min, followed by washing with 1% acetic acid three times and drying in air. Then, 150 µL of TRIS (10 mM) was used to dissolve SRB stain protein bounds. The absorbance was measured at 540 nm using a BMGLABTECH®- FLUOstar Omega microplate reader (Ortenberg, Germany).

### In-vitro Antibacterial activity tests

#### Inoculum standardization for sensitivity tests

The bacterial inoculums were adjusted photometrically using a spectrophotometer at 625 nm to give absorbance from (0.08–0.1) to approximately (1.5 × 10^8^) CFU/ml equal to 0.5 McFarland’s standard [29] to be used in agar sensitivity and MIC assays.

#### Antimicrobial susceptibility test (AST) assay

The antibacterial activity of both AG-Zn and Cu NPs against inoculated agar with the identified *E. coli* strains, including O17, O78, O91, O121, and O159, was assessed using the agar well diffusion method [30]. A sterile cork borer of 6 mm in diameter was used to make the wells. Subsequently, about 100 µL of Zn and Cu NPs (at concentrations of 20, 40, 60, 80, and 100 mg/ml) were filled into respective wells. After incubation at 37 °C for 24 h, the inhibition zone diameters (IZD) were measured in millimeters (mm). In addition, AST of various antibiotics, including amoxiclav AMC (30 µg), cefotaxime CTX (30 µg), gentamicin GEN (10 µg), doxycycline (30 µg), sulphamethoxazole-trimethoprim (Cotrimoxazole) COT (25 µg) and enrofloxacin ENR (5 µg) from six different families of commercial antibiotics β-lactamases-inhibitors, cephalosporins, aminoglycosides, tetracyclines, folates-inhibitors, and quinolones was performed using disk diffusion method to judge the susceptibility patterns and IZD of the used drugs. Results were classified as susceptible, intermediate, or resistant by measuring IZD [31]. Each antimicrobial assay was performed simultaneously in triplicate.

#### MIC and MBC values

The MIC as a quantitative bioassay was determined by using a micro-broth dilution test in a 96-well microplate using standard guidelines of CLSI [31] against the identified *E. coli* strains; O17, O78, O91, O121, and O159. A 60 mg/ml solution of CuNPs was twofold serially diluted to 0.127 µg/ml. Both negative (only drug without bacteria) and positive (only bacteria without drug) control wells were prepared. Referring to the results of the MIC assay, a loopful from each clear well was streaked on the EMB agar, incubated at 37 °C/24 h, and then observed for growth. The lowest concentration that showed no turbidity was recorded as the MIC, and the lowest concentration that did not show any growth was recorded as the minimum bactericidal concentration (MBC). The experiment was performed in duplicate. In addition, TEM was used to study the antibacterial effect of AG-CuNPs on *E. coli* O17 cultures after incubation with 50 mg/ml of CuNPs at 37 °C/overnight, then processed and observed in JEOL 1400 (JEOL, Ltd., Japan) running at 80 kV [32].

#### Antibacterial performance of AG-CuNPs against E. coli O17 strain

The effect of time and concentration of AG-CuNPs on its antibacterial performance against *E. coli* O17 were assessed as mentioned Li et al. [33]. *E. coli* O17 was prepared in approximately 1 × 10^8^ CFU/ml, and then 50 µl of this bacterial suspension was inoculated into 950 µl of various concentrations of AG-CuNPs. After several sterilization times, these grown bacterial suspensions were inoculated on EMB Petri dishes. This study used various times of (*E. coli* O17- AG-CuNPs) contact at intervals from 5 to 30 min with various concentrations of AG-CuNPs ranging from 5 to 60 mg/ml). Finally, all plates were incubated at 37 °C/24 h, and the number of surviving cells was counted. The number of surviving cells was calculated as a percentage compared to the positive control plates without AG-CuNPs; then, results were subtracted from 100% to obtain the antibacterial rate. Each experiment was performed in duplicate and mean values were calculated.

*Detection of the efficacy of CuNPs on virulence genes expression of E. coli O17 strain by real-time quantitative PCR (qRT–PCR)*:

Pure *E. coli* O17 strain at a dose of 10^8^ CFU/ml (the experimental challenge dose) was mixed with CuNPs at a dose of 50 mg/ml broth and incubated at 37 °C overnight, then subjected to qRT–PCR using comparative cycle threshold (CT) to detect the fold change value of the different target virulence genes expression. The treated *E. coli* strain sample containing (*ibeA, hlyA, iss, and papC*) was compared with the control non-treated *E. coli* strain sample. In addition, the relative expression of the target genes was determined in comparison to a standard reference gene of 16 S- rRNA. RNA extraction from samples (treated *E. coli* strain isolate and control non-treated *E. coli* strain isolate) was performed using QIAamp RNA mini kit instructions when 200 µl of the sample were added to 600 µl RLT buffer containing 10 µl β-mercaptoethanol per 1 ml, incubated at room temperature for 10 min. One volume of 70% ethanol was added to the cleared lysate, and these steps was completed according to the Purification of total RNA protocol of the QIAampRNeasy Mini kit (Qiagen, Germany, GmbH). Primers were utilized in a 25- µl reaction containing 10 µl of the 2x HERA SYBR® Green RT-qPCR Master Mix (Willowfort, UK), 1 µl of RT Enzyme Mix (20X), 0.5 µl of each primer of 20 pmol concentration, 5 µl of water, and 3 µl of RNA template. Amplification curves and ct values were determined by the step one software Primers sequences, target genes, amplicon sizes and cycling condition were showed in the table Table 1. The CT of the treated sample was compared with that of the control non-treated sample to estimate the gene expression variation on the treated sample’s RNA according to the “ΔΔCt” method by using the following ratio: (2^−ΔΔCt^). Each assay was performed simultaneously in triplicate [34].

### In-vivo Assessment of the Cu NPs’ antimicrobial efficacy

#### Experimental design

A total of 90 one-day-old broiler chicks were purchased from a private farm. Then, the livers of 10 chicks were tested bacteriologically to ensure they were free from any systemic *E. coli* infection. The remaining 80 chicks were divided into four groups (20 chicks /group); G1: negative control, G2: positive control infected orally with *E. coli* O17 (1 × 10^8^ CFU/ml), G3: non-infected treated with (AG-CuNPs 50 mg/kg body weight) and G4: infected treated with (AG-CuNP 50 mg/kg body weight). This effective dose of AG-CuNPs (50 mg/kg body weight) was adjusted from our *in-vitro* sensitivity tests, TEM, and cytotoxicity assays. All groups had the same management and vaccination with free access to Feed and water. Two weeks old broilers were infected and then observed daily for morbidity, mortality, clinical signs, and necropsy finding. All treatments were administered following the onset of clinical symptoms on the 20th and continued for five successive days via drinking water until the 24th day. The experiment was conducted until the 35th day of age. The animal studies were approved by Research Ethics Committee for environmental and clinical studies (Protocol number: 165,429) at Animal Health Research Institute (AHRI) and were carried out under Egyptian Ethics Committee Guidelines and the NIH guidelines for the Care and Use of Laboratory Animals. All animal experiments were performed following the ARRIVE guidelines (https://arriveguidelines.org).

#### Sampling

##### **Blood samples**

Blood samples were collected from the wing vein (5 broilers/group) in a centrifuge tube without anticoagulant to get the serum on the 25th and 35th day related to after treatment and at the end of the study respectively. Alanine aminotransferase (ALT) [35], gamma-glutamyl transferase (GGT) [36], alkaline phosphatase (ALP) activities [37], total protein (TP) [38], and albumin [39] were determined. Globulin was detected by subtracting the albumin value from TP. For immunological evaluation, serum lysozyme activity was measured by agarose gel lysis assay [40]. The C-reactive protein, one of the anti-inflammatory parameters, was determined [41]. Uric acid [42] and creatinine [43] were estimated to evaluate kidney functions. All tests were determined using commercial kits (Spectrum and EgyChem, Egypt).

##### **Tissue samples**

Liver (1 g) and intestine (1 g) from all infected groups (3 broilers/group) were collected after treatment under aseptic conditions for *E. coli* re-isolation and count. First, tissues were transferred to 9 ml phosphate buffer saline (PBS) to prepare the initial suspension. Subsequently, tenfold serial dilutions were made, and then 0.1 ml of each dilution was spread on EMB agar plates and incubated aerobically at 37 °C/24 h. Then the number of survived cells was counted. For detection of Cu residues; tissue specimens from the liver (as an edible organ), breast, and thigh muscles were collected (5 broilers/group) after treatment and at the end of the study. Residues were measured using atomic absorption spectrophotometer (AA-7000 – Shimadzu) according to the method described by Okoye et al., [44]. A schematic illustration of the experimental design were showed in Supplementary Fig. 1.

### Statistical analysis

One-way analysis of variance (ANOVA) was applied for statistical analysis. Duncan multiple range post-hoc analysis tests using IBM SPSS software statistical program (version 20.0) were used to compare means at the significance level of P < 0.05. Data were expressed as mean ± SE (standard error) and N = 5.

## Results

### Incidence of E. coli isolates among examined samples

After microscopically gram staining testing, biochemical identification, and confirmation of suspected pure isolates, *E. coli* was isolated with a total incidence rate of 20% (20/100) in all examined samples collected from diseased broilers, 18% ( 9/50) from liver samples, and 22% (11/50) from intestinal samples.

### Serological typing and characterization of E. coli isolates

Results revealed that the examined samples belonged to O17: H18, O121: H7, O78, O127: H6, O91: H21, O103: H2 and O159. O78 was the most prevalent serotype with 30%, followed by O91: H21 with 20%, followed by O17: H18 with 18%. O17 was isolated and detected only in liver samples, whereas O78 and O91 were isolated from liver and intestinal samples, and O121 and O159 were isolated from intestinal samples only.

### Molecular detection of the virulence genes using uniplex cPCR

**Fig. 1:**
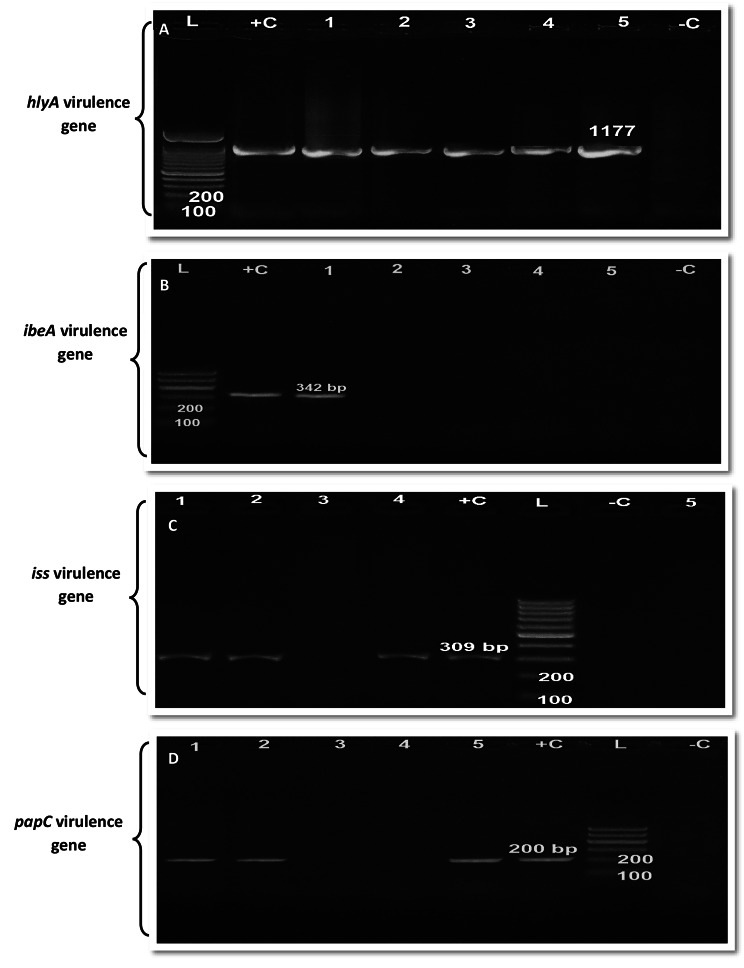
Grouping of 4 separate images for (**A**) Agarose gel electrophoresis for PCR amplification of *hlyA* virulence gene at (1177 bp), (**B**) *ibeA* virulence gene at (342 bp), (**C**) *iss* virulence gene at (309 bp), (**D**) *papC* virulence gene at (200 bp) in APEC serotypes: Lane L (ladder), Lane + C (control positive), Lane - C (control negative), Lane 1 (serotype O17), Lane 2 (serotype O78), Lane 3 (serotype O91), Lane 4 serotype (O121) and Lane 5 (serotype O159)

In this study work we used uniplex conventional PCR to avoid false negative results during gel electrophoresis interpretation as some gene amplification may give bands at nearly closed molecular weight, so uniplex PCR is more accurate than multiplex PCR. The *hlyA* gene was expressed in all serotyped APEC isolates. In contrast, the *ompA* gene was not expressed in all serotyped APEC isolates. However, four virulence genes, *ibeA, hlyA, iss*, and *papC*, were expressed in only the O17 serotype. The results are shown in Tables 2 and Fig. 1A-D. Individual Agarose gel electrophoresis images for PCR amplification of all virulence genes were showed in supplementary Figs. 2–5.

**Table 2:** Molecular detection of the virulence genes using uniplex cPCR

Serotyped isolate	*ibeA*	*hlyA*	*iss*	*papC*	*ompA*
O 17	**+**	**+**	**+**	**+**	**-**
O 78	**-**	**+**	**+**	**+**	**-**
O 91	**-**	**+**	**-**	**-**	**-**
O 121	**-**	**+**	**+**	**-**	**-**
O 159	**-**	**+**	**-**	**+**	**-**

### Characterization of AG-CuNPs and ZnNPs

#### Particle size and zeta potential

**Fig. 2:**
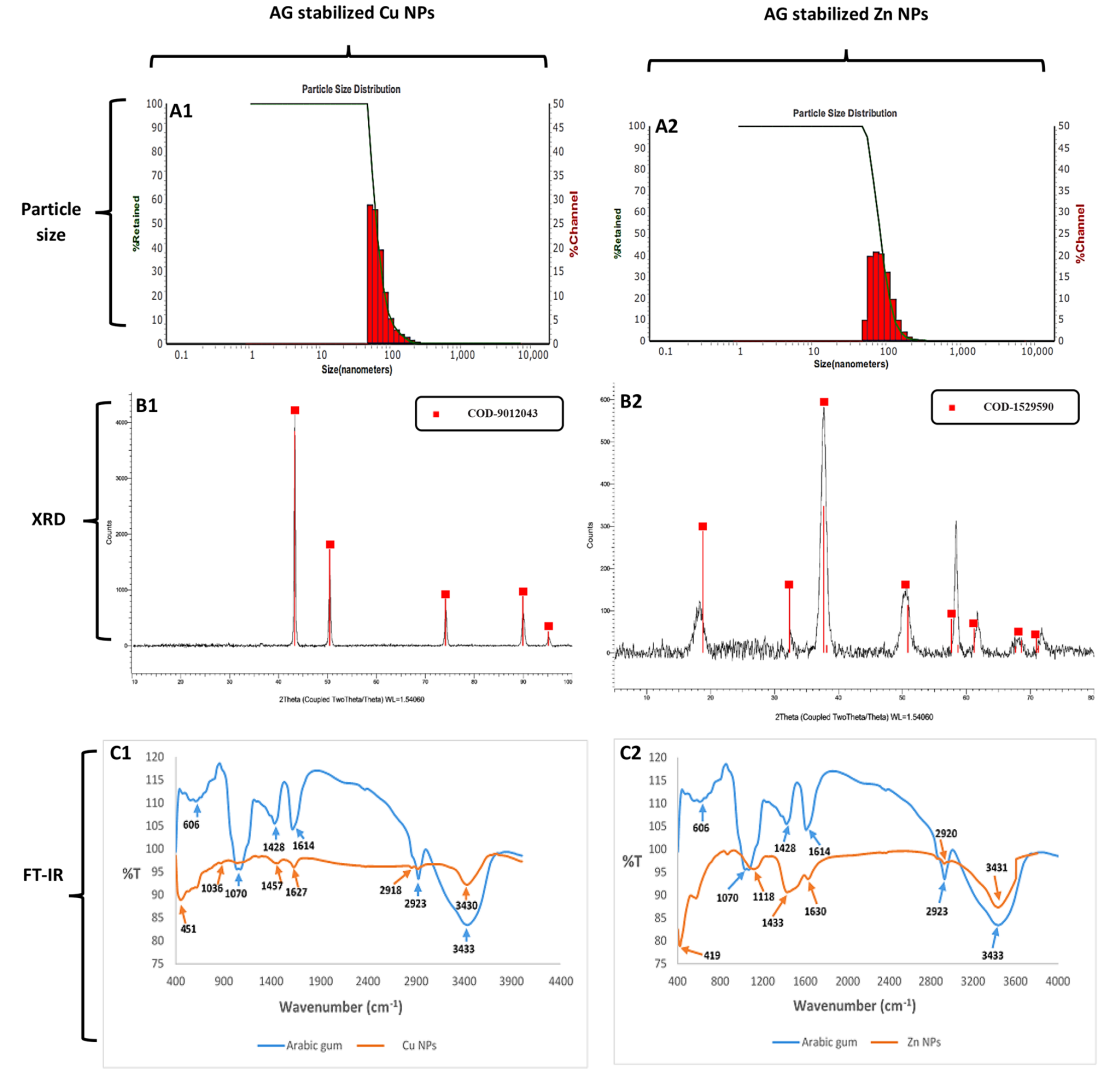
Characterization of AG stabilized Cu and ZnNPs. (**A1** and **A2**) particle size pattern of synthesized NPs showing (**A1**) size distribution of CuNPs of 58 nm, (**A2**) size distribution of ZnNPs of 75 nm. (**B1** and **B2**) XRD pattern of Cu and ZnNPs respectively at the wavelength of 1.54060. (**C1** and **C2**) FT-IR spectra of Cu and ZnNPs respectively at the scanning range of 4000 − 400 cm^− 1^ compared with Arabic gum

The results of Zetasizer showed that the average particle size of Cu and Zn NPs was58nm and 75 nm, respectively, with a narrow size distribution; poly dispersity index (PDI) was0.16 and 0.15, respectively, and surface charges of-24.8 and − 25.5 mV, respectively (Fig. 2A1 and A2).

#### X-ray diffraction studies

The XRD pattern of synthesized AG-Cu and ZnNPs is shown in Fig. 2B1 and B2. For the CuNPs, prominent peaks can be observed at 2θ of 43.3, 50.4, 74.1, 89.9, and 95.1°. The assignments of 2θ position and other parameters for AG-CuNPs from XRD agree with the reference code (COD-9,012,043) at a wavelength of 1.54060 with a cubic crystal system and space group Fm-3 m.For the AG-Zn NPs, prominent peaks can be observed at 2θ of 18.81, 32.34, 38.15, 50.86, 57.68, 61.23,68.64, and 70.96°.The assignments of 2θ position and other parameters for ZnNPs from XRD agree with the reference code (COD-1,529,590) at a wavelength of 1.54060 with a hexagonal crystal system and space group P-3m1.

#### FT-IR spectra

FT-IR spectra of *Arabic gum* synthesized and stabilized AG-Cu, and ZnNPs are shown in (Fig. 2; C1 and C2). For Arabic gum, the peak at 3433 cm^− 1^ showed the presence of the N-H bond in stretching mode, and the peak at 2923cm^1^ showed the presence of the O-H bond in stretching mode (carboxylic acid).The peak at 1614 cm^− 1^referred to the presence of the N-H bond, which bent in primary amine. The peak at 1428 cm^− 1^showed the presence of the C-O-H bond in the bending mode, while the peaks at 1070 cm^− 1^and 606 cm^− 1^showed the presence of C-O and C-l bondsin the stretching mode. For AG-CuNPs, the N-H bond in stretching mode is represented at 3430 cm^− 1^, while the peak at 2918 cm^− 1^ showed the presence of O-H bond stretching mode (carboxylic acid). Furthermore, the peak at1627cm^− 1^referredto the presence of the N-H bond, which bended in primary amine, while the peak at 1457 cm^− 1^referredto the presence of the N-H bond, which bent in secondary amine. The peaks at 1036 cm^− 1^and 451 cm^− 1^ showed the presence of the C-O and C-l bond in the stretching mode.

For AG-ZnNPs, the N-H bond in stretching mode is represented at the peak of 3431 cm^− 1^, while the peak at 2920 cm^− 1^revealedthe presence of O-H bond stretching mode (carboxylic acid).In addition, the peak at 1630 cm^− 1^ referred to the presence of the N-H bond, which bent in primary amine, the peak at 1433 cm^− 1^ showed the presence of the C-O-H bond in the bending mode, the peak at 1118 cm^− 1^ showed the presence of C-O bond in stretching mode, and the peak at 419 cm^− 1^ showed the presence of C-l bond in stretching mode.

#### Morphological characters and cytotoxicity assay

**Fig. 3:**
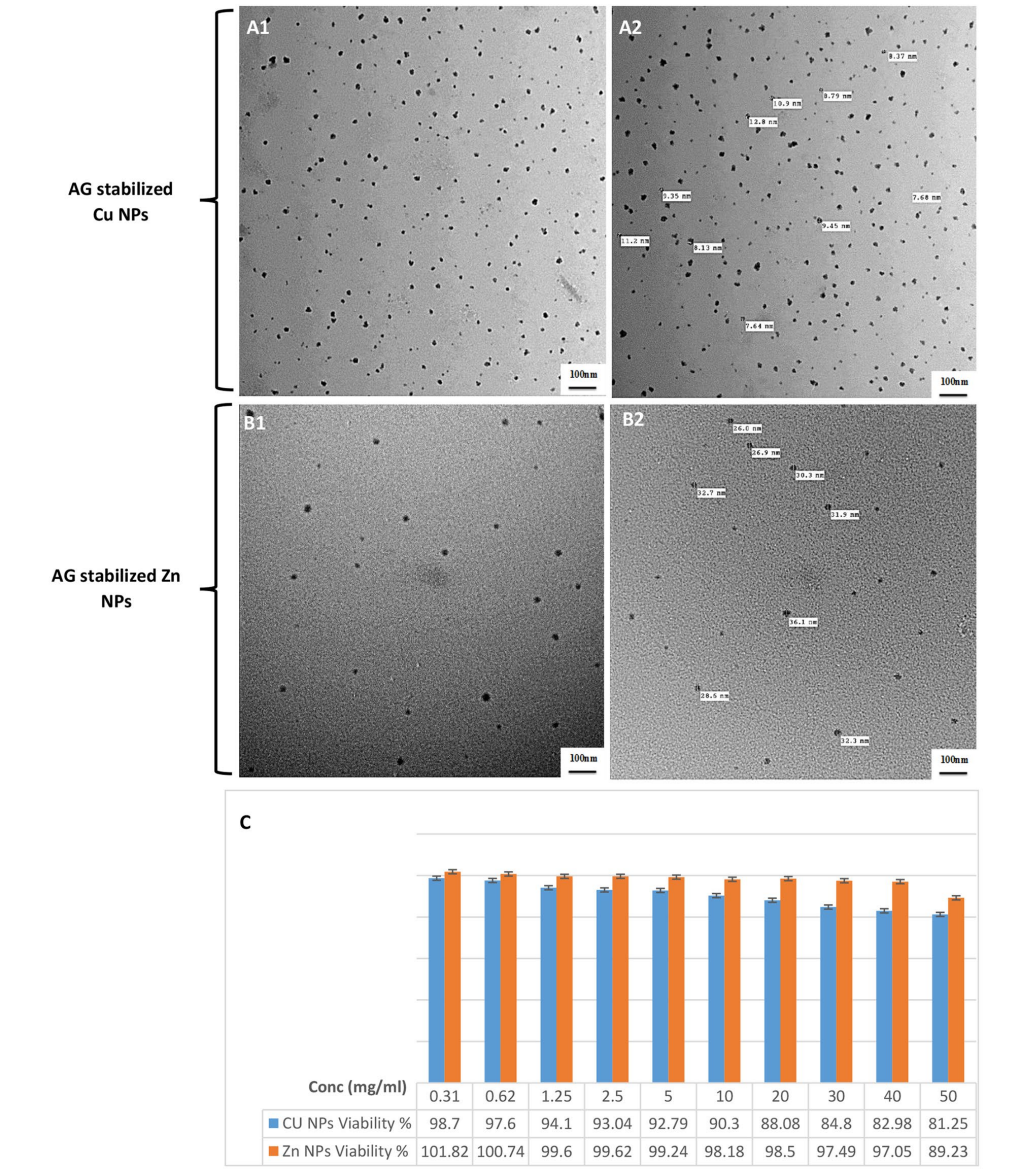
Morphological characters and cytotoxicity assay of AG stabilized Cu and ZnNPs, (**A1** and **A2**) is TEM imaging of AG stabilized CuNPs showed well dispersed nanosphere particles and size ranged from 7.68 to 19.5 nm, Scale bar = 100 nm. (**B1** and **B2**) is TEM imaging of AG stabilized ZnNPs showed spherical nanoparticles without agglomeration and size ranged from 23.4 to 39.6 nm, Scale bar = 100 nm. (**C**) is SRB cytotoxicity assay of AG stabilized Cu and ZnNPs at different concentrations ranged from 0.31 to 50 mg/ml

TEM images of stabilized AG-Cu and ZnNPs (Fig. 3; A1, 2 and B1, 2) showed distinctive single NPs with spherical shapes without aggregation. Their size ranged from 7.68 to 19.5 nm for CuNPs (Fig. 3; A1 and A2) and 23.4 to 39.6 nm for AG-ZnNPs (Fig. 3; B1 and B2).

In the SRB assay, the survival rate was decreased from 98.7to 81.25% at concentrations of 0.31and 50 mg/ml, respectively, as shown in Fig. 3C for the cells incubated with different concentrations of AG-CuNPs. However, the survival rate was decreased from 101.82 to 89.23% at concentrations of 0.31 and 50 mg/ml, respectively, for the cells incubated with different concentrations of AG-ZnNPs, as shown in Fig. 3C. NPs did not show toxicity concentrations less than 50 mg/ml, and cells kept the same morphological characteristics as control ones.

### In-vitro Antibacterial activity tests

#### AST, MIC, and MBC of AG-CuNPs against tested E. coli isolates

**Fig. 4:**
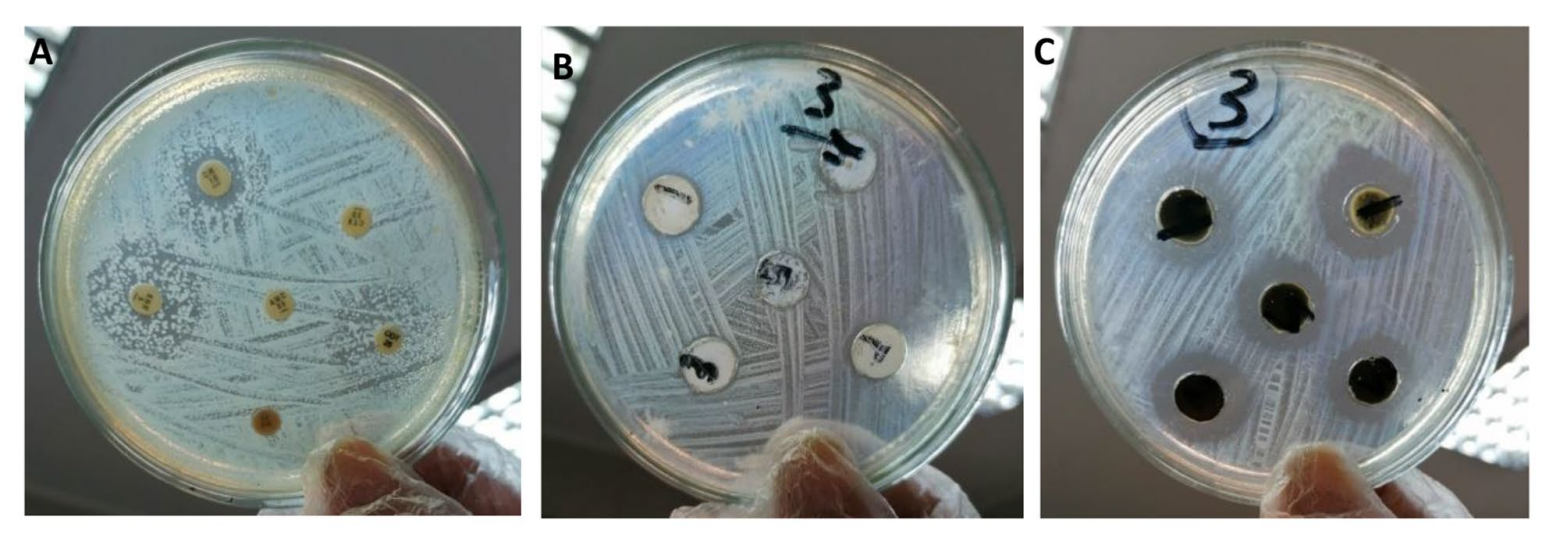
Antimicrobial sensitivity tests (AST) of *E. coli* O17 strain (**A**) against commercial antibiotic disks indicated complete resistance and (**B**) against various AG-ZnNPs concentrations indicated complete resistance, while (**C**) against AG-CuNPs showed various inhibition zone diameters

AST of commercial antibiotics or AG-ZnNPs indicated the complete resistance of all *E. coli* tested strains to all tested antibiotics and all of the used AG-ZnNPs concentrations from 20 to 100 mg/ml. Whereas AST of AG-Cu NPs (Table 3) revealed a significant antibacterial activity with markedly ascending IZDs against all *E. coli* tested strains corresponding to the used concentrations from 20 to 100 mg/ml. In most of the detected strains, no significant difference was recorded between IZDs of both (20 and 40) mg/ml or (80 and 100) mg/ml; for example, the AST of the *E. coli* O17 strain used in our *in-vivo* study (Fig. 4; A-C). The *E. coli* O17 strain was reported to have the highest IZD ranging from 19.66 ± 0.33 to 25.00 ± 0.00 mm, corresponding to the used concentration. The IZDs of AG-CuNPs reflected effective antibacterial activity that is similar to other traditional antimicrobials. Results of MIC and MBC of CuNPs varied according to the *E. coli* tested strains. MIC values were 3.75 mg/ml for O78, 7.5 mg/ml for O17, and 15 mg/ml for O91, O121, and O159, while the bactericidal effect of AG-CuNPs (MBC) was reported at 60 mg/ml well for O17, O78, and O121 only, while other O91 and O159 required higher concentrations than 60 mg/ml.

**Fig. 5:**
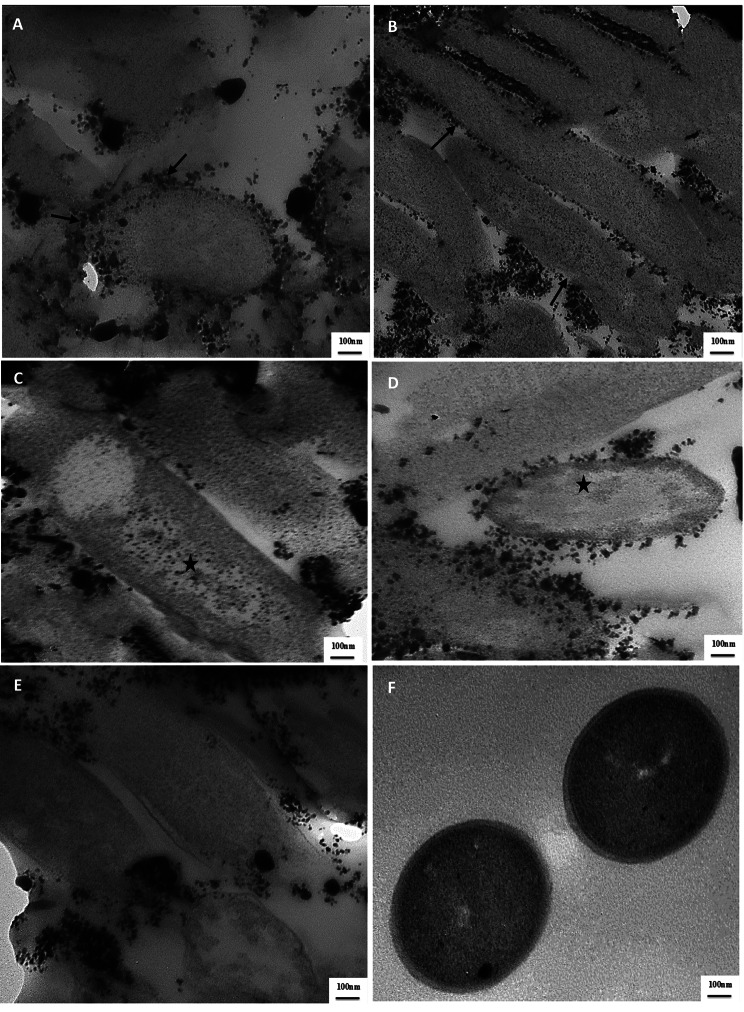
TEM images of *E. coli* O17 cells grown in the media containing 50 mg/ml of AG stabilized CuNPs showed accumulation of nanoparticles on the cell wall (arrows) and attack of nanoparticles (**A**), (**B**) showed elongation of bacterial cell and particles around bacterial surface (arrows). (**C**) showed internalization of NPs intracellular and distribution in cytoplasm (star). (**D**) showed ghost appearance of cell with empty and flaccid structure (star) and cytoplasm with low electronic density. (**E**) showed disruption of cell wall and cellular damage 24 h post incubation compared with control bacterial cells only (**F**). Scale bar = 100 nm

Furthermore, the bactericidal activity of AG-CuNPs on the *E. coli* O17 strain was confirmed using TEM analysis (Fig. 5; A-F),where anchoring and accumulating NPs around bacterial cell wall were recorded besides the intracellular distribution of NPs in the bacterial cytoplasm giving ghost appearance that ended with cell damage.

**Table 3:** The inhibition zone diameters of AG-Cu NPs on isolated *E. coli* strains

Strains	Inhibition zone diameters in millimeter at various AG-Cu NPs concentrations				
	20 mg/ml	40 mg/ml	60 mg/ml	80 mg/ml	100 mg/ml
**O17**	19.66 ± 0.33 ^a^	20.00 ± 0.00 ^a^	20.00 ± 0.00 ^a^	22.33 ± 0.33 ^b^	25.00 ± 0.00 ^c^
**O78**	17.00 ± 0.57 ^a^	18.00 ± 0.00 ^a^	20.00 ± 0.00 ^b^	22.00 ± 0.57 ^c^	24.00 ± 0.57 ^d^
**O91**	16.66 ± 0.33 ^a^	17.00 ± 0.00 ^a^	18.33 ± 0.33 ^b^	21.00 ± 0.00 ^c^	22.33 ± 0.33 ^d^
**O121**	18.00 ± 0.00 ^a^	19.00 ± 0.57 ^a^	21.66 ± 0.33 ^b^	22.00 ± 0.00 ^bc^	23.00 ± 0.57 ^c^
**O159**	16.33 ± 0.33 ^a^	18.00 ± 0.00 ^b^	18.00 ± 0.57 ^b^	20.33 ± 0.33 ^c^	21.00 ± 0.57 ^c^

The various letters in the same row indicate statistically significant differences when (P < 0.05) (Mean ± SE) n = 3

#### Antibacterial performances of AG-Cu NPs on E. coli O17 strain

**Fig. 6:**
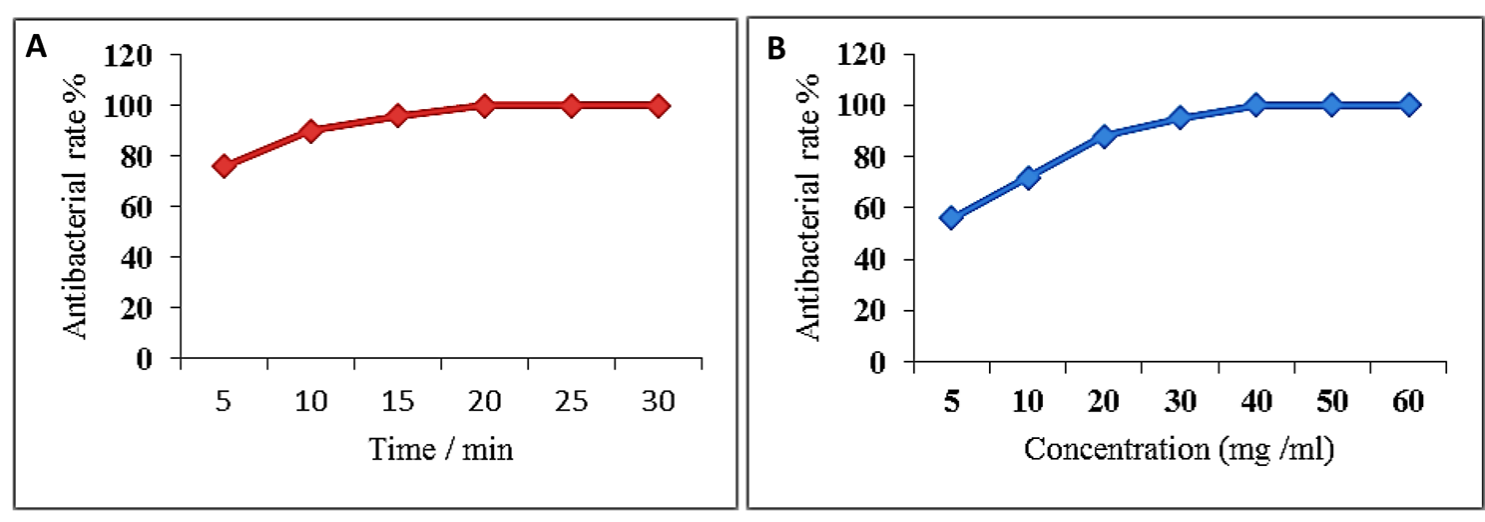
(**A**) The antibacterial rates of AG-CuNPs against *E. coli* O17 isolate post various times of contact, (**B**) The antibacterial rates of AG-CuNPs against *E. coli* O17 on various concentrations

Figure 6 displays the antibacterial effect of AG-CuNPs on the viability of *E. coli* O17 post-incubation with different contact times or different AG-CuNPs concentrations to meet the bactericidal activity threshold. As shown in Fig. 6A, the antibacterial rate of AG-CuNPs against *E. coli* O17 was detected from 5 to 30 min, which increased with time. The bactericidal effect appeared at 20 min when the antibacterial rate reached 100%. The effect of AG-CuNPs concentrations on *E. coli* O17 inactivation has been illustrated In Fig. 6B. Thus, the antibacterial rate was 100% at 40 mg/ml AG-CuNPs. The surviving bacteria with AG-CuNPs were significantly less than the control groups throughout all times and concentrations.

#### Efficacy of AG-CuNPs on target virulence genes expression of E. coli O17 strain by real-time quantitative PCR (RTQ – PCR)

**Fig. 7:**
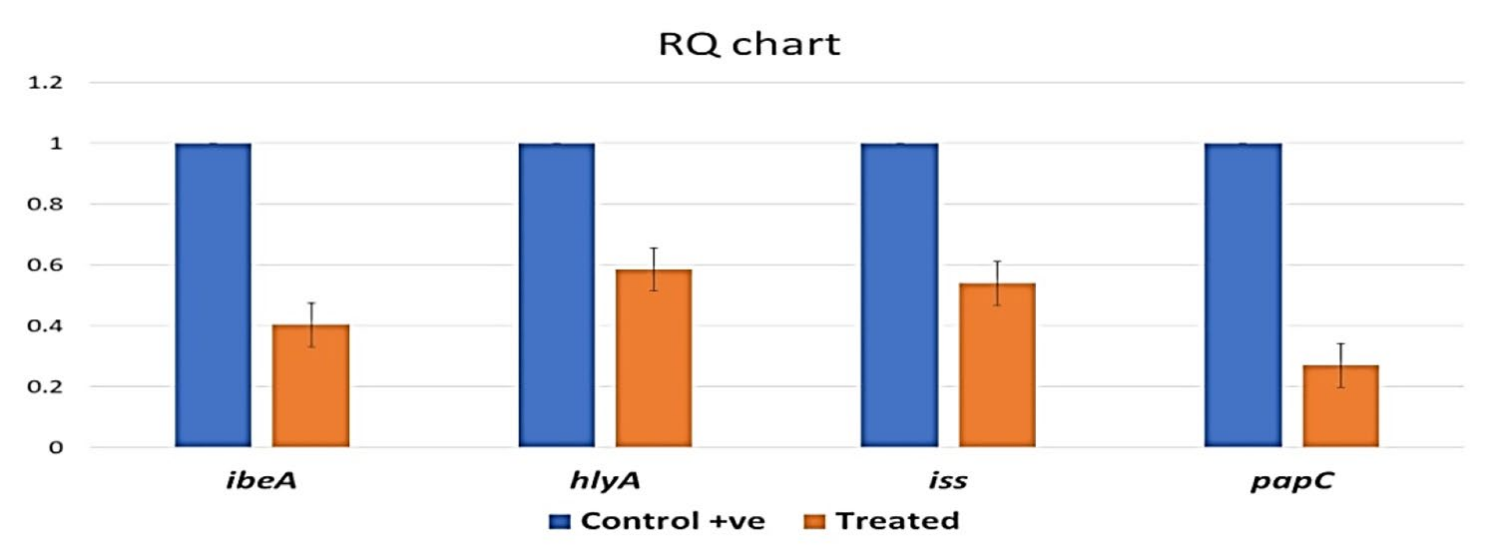
Results of expression and fold change values of target virulence genes in *E. coli* samples of that treated with AG-CuNPs in relation to the genes in control non-treated sample by RTQ – PCR showed a significant difference in all of these detected genes

The expression of virulence genes was significantly down regulated in the treated *E. coli* compared to the control non-treated sample with the treatment dose of AG-CuNPs (50 mg/ml). The *papC* gene was mostly down regulated with 0.2698, while the *ibeA*, *iss*, and *hlyA* genes were down regulated with 0.403320, 0.5396, and 0.586 respectively, as shown in Fig. 7.

### In-vivo Assessment of the AG-Cu NPs antimicrobial efficacy

#### Clinical signs, post-mortem findings, and mortalities

Clinical signs of *E. coli* appeared on the 4th and 5th days post-infection as anorexia, depression, ruffled feathers, and diarrhea. No signs were reported in the negative control G1. Five chicks died in the infected control G2 post-inoculation and throughout the experiment where their post-mortem lesions in most carcasses displayed severe to a mild degree of visceral congestion, enlargement, congestion of liver and spleen, mild pericarditis, and precipitated urea in the ureter. Conversely, no mortality was recorded in other negative control or treated groups. As well, general health and behavior activities showed a noticed improvement post-treatment.

#### E. coli re-isolation and count

No *E. coli* were re-isolated from the liver of non-infected G1 or G3, indicating the absence of systemic infection. In the infected control G2, *E. coli* counts were (3.67 × 10^3^ ± 0.33) and (50.33 × 10^3^ ± 2.03) CFU/g in liver and intestine samples, respectively. Post-treatment, no *E. coli* was re-isolated from liver samples of infected treated G4, while its count in intestine samples was 2 × 10^3^ ± 0.58 CFU/g.

#### Serum biochemical parameters

TP, albumin, and globulin levels were significantly decreased, while ALT, GGT, and ALP activities were significantly increased in infected control G2 compared to control G1 (Table 4). Post-treatment, TP, albumin, and globulin were non-significantly changed in non-infected treated G3 compared to G1. However, the activities of enzymes were significantly increased in non-infected treated G3 compared to G1, while at the end of the experiment, results returned to their normal levels. There were significant increases in TP, albumin, and globulin besides significant decreases in most of the detected enzymes’ activities in infected treated G4 than G2. In contrast, it showed no significant difference from G1 in most of these detected parameters at the end of the experiment.

**Table 4:** Effect of AG-Cu NPs on some serum biochemical parameters

G	Periods	Total protein (g/dl)	Albumin (g/dl)	Globulin (g/dl)	ALT (u/l)	GGT (u/l)	ALP (u/l)
G1	**PT**	3.83 ± 0.12 ^c^	1.54 ± 0.04 ^b^	2.29 ± 0.08 ^c^	52.95 ± 1.11 ^a^	16.73 ± 0.51 ^a^	61.29 ± 1.67 ^a^
	**AEE**	4.07 ± 0.11 ^bc^	1.70 ± 0.02 ^b^	2.37 ± 0.11 ^ab^	52.82 ± 0.86 ^a^	16.91 ± 0.62 ^a^	63.26 ± 1.92 ^a^
G2	**PT**	3.22 ± 0.08 ^a^	1.19 ± 0.06 ^a^	2.02 ± 0.02 ^a^	63.36 ± 0.91 ^c^	21.33 ± 0.86 ^c^	70.36 ± 0.73 ^c^
	**AEE**	3.55 ± 0.07 ^a^	1.46 ± 0.04 ^a^	2.09 ± 0.04 ^a^	58.71 ± 0.69 ^b^	20.56 ± 0.42 ^c^	68.71 ± 0.81 ^b^
G3	**PT**	3.75 ± 0.06 ^bc^	1.52 ± 0.04 ^b^	2.23 ± 0.03 ^bc^	57.45 ± 0.74 ^b^	18.49 ± 0.44 ^ab^	63.49 ± 0.95 ^ab^
	**AEE**	4.21 ± 0.10 ^c^	1.73 ± 0.04 ^b^	2.49 ± 0.09 ^b^	54.45 ± 1.30 ^a^	17.22 ± 0.44 ^ab^	62.00 ± 0.40 ^a^
G4	**PT**	3.55 ± 0.04 ^b^	1.44 ± 0.06 ^b^	2.11 ± 0.05 ^ab^	56.19 ± 0.65 ^b^	19.80 ± 0.81 ^bc^	65.82 ± 1.12 ^b^
	AEE	3.91 ± 0.03 ^b^	1.73 ± 0.05 ^b^	2.18 ± 0.02 ^a^	53.18 ± 0.80 ^a^	18.61 ± 0.39 ^b^	62.49 ± 0.47 ^a^

The various letters in the same colon of the same period indicate statistically significant differences when (P < 0.05). G1 = negative control, G2 = infected control, G3 = noninfected + AG-CuNPs, G4 = infected + AG-CuNPs, PT = Post treatment, AEE = At experimental end (Mean ± SE) n = 5

#### Immunological, Anti-inflammatory, and kidney function biomarkers

The lysozyme activity was significantly decreased, while C-reactive protein, uric acid, and creatinine levels were significantly increased in infected control G2 compared to control G1 (Table 5). Post-treatment and at the end of the experiment, the lysozyme activity was significantly increased, while C-reactive protein was significantly decreased with no significant effect on uric acid or creatinine in both non-infected treated G3 and infected treated G4 compared to control G1. The lysozyme activity was significantly increased, while C-reactive protein, uric acid, and creatinine levels were significantly decreased in the infected treated G4 compared to infected control G2 (Table 5).

**Table 5:** Effect of AG-Cu NPs on lysozyme activity, C reactive protein, uric acid and creatinine

G	Periods	Lysozyme activity (µMol)	C reactive protein (mg/l)	Uric acid (mg/dl)	Creatinine (mg/dl)
G1	**PT**	120.61 ± 1.93 ^b^	4.98 ± 0.11 ^b^	5.91 ± 0.25 ^a^	0.43 ± 0.01 ^a^
	**AEE**	122.41 ± 1.52 ^b^	4.09 ± 0.22 ^b^	5.87 ± 0.07 ^a^	0.51 ± 0.01 ^a^
G2	**PT**	107.80 ± 1.66 ^a^	28.11 ± 0.41 ^d^	7.18 ± 0.19 ^b^	0.56 ± 0.02 ^c^
	**AEE**	112.46 ± 1.80 ^a^	14.21 ± 0.26 ^c^	6.88 ± 0.20 ^b^	0.58 ± 0.01 ^b^
G3	**PT**	132.99 ± 1.59 ^c^	4.01 ± 0.09 ^a^	6.10 ± 0.38 ^a^	0.44 ± 0.02 ^ab^
	**AEE**	126.93 ± 1.81 ^b^	3.07 ± 0.04 ^a^	5.52 ± 0.25 ^a^	0.52 ± 0.01 ^a^
G4	**PT**	129.11 ± 1.65 ^c^	10.22 ± 0.28 ^c^	6.63 ± 0.32 ^ab^	0.49 ± 0.01 ^b^
	AEE	125.89 ± 1.27 ^b^	4.08 ± 0.05 ^b^	5.66 ± 0.37 ^a^	0.51 ± 0.02 ^a^

The various letters in the same colon of the same period indicate statistically significant differences when (P < 0.05). G1 = negative control, G2 = infected control, G3 = noninfected + AG-CuNPs, G4 = infected + AG-CuNPs, PT = Post treatment, AEE = At experimental end (Mean ± SE) n = 5

#### Residues of Cu in liver and muscles

In Table 6, results of Cu residues in the liver showed a significant increase in G2 than G1. After treatment and at the end of the experiment, all treated groups G3 and G4 were significantly more than control groups G1 and G2. In muscle, Cu residues showed a significant increase in G2 than G1. After treatment, all treated groups G3 and G4 were significantly more than control groups G1 and G2, while at the end of the experiment, all treated groups revealed no significant difference from G1.

**Table 6:** Effect of AG-Cu NPs on copper (Cu) residues in liver and muscle

Periods	G	G1	G2	G3	G4
After treatment	**Liver Cu (mg/kg)**	1.71 ± 0.02 ^a^	2.09 ± 0.05 ^b^	2.26 ± 0.04 ^b^	3.10 ± 0.13 ^c^
	**Muscle Cu (mg/kg)**	0.24 ± 0.00 ^a^	0.24 ± 0.01 ^a^	0.27 ± 0.00 ^b^	0.33 ± 0.00 ^c^
At the end of the experiment	**Liver Cu (mg/kg)**	1.49 ± 0.02 ^a^	1.59 ± 0.03 ^a^	1.80 ± 0.02 ^b^	2.45 ± 0.05 ^c^
	**Muscle Cu (mg/kg)**	0.20 ± 0.00 ^b^	0.23 ± 0.01 ^c^	0.18 ± 0.00 ^b^	0.20 ± 0.00 ^b^

The various letters in the same row of the same period and organ indicate statistically significant differences when (P < 0.05). G1 = negative control, G2 = infected control, G3 = noninfected + AG-CuNPs, G4 = infected + AG-CuNPs, PT = Post treatment, AEE = At experimental end (Mean ± SE) n = 5

## Discussion

APEC is an infectious bacterium causing colibacillosis. Colisepticemia is the most common form of colibacillosis, which is still a significant threat to the poultry industry, causing high economic losses due to mortality and condemnations, and is a widespread disease [7]. The expansion of bacterial-resistant strains to different antimicrobials has encouraged researchers to search for other antimicrobial options. The antibacterial potentials of MONPs may introduce a probable solution to this problem. Recently, non-toxic origin-mediated synthesis of AG-CuNPs has been one of the critical roles of therapeutic bio applications. Therefore, our study was designed to compare the possible antibacterial effect of AG-Cu and Zn NPs against various field-isolated APEC strains and their virulence genes *in-vitro*. Green synthesized AG-CuNPs as an antibiotic alternative were used besides analyzing their effects *in-vivo* against *E. coli* O17 infection in broilers on clinical signs, *E. coli* re-isolation and count, immune status, anti-inflammatory activity, liver and kidney biomarkers, and muscle and liver tissues residues.

APEC, the main causal agent of colibacillosis in poultry farms, can induce enteric and extraintestinal infections in a syndrome associated with diarrhea and/or enteritis [45, 46]. APEC can colonize the intestinal and respiratory tracts of chickens [47]. Hussain et al., [48] isolated APEC from fecal samples of broilers showing signs of colibacillosis. As well, Saha et al., [49] reported that broiler droppings had the highest amount of APEC isolates (33.33%) followed by liver (19.54%). Abd El Tawab et al., [50] isolated APEC with higher rates of isolation from intestine (31.3%), liver (28.1%) followed by other organs. In the present study, *E. coli* was isolated from diseased broilers with an average incidence rate of 20%. Our results are nearly parallel to the results obtained by El-Seedy et al. [51], who reported a prevalence rate of 23%, while Hasan et al. [52] stated a rate of 29%. A high prevalence rate from diseased broilers (37.1%) was recorded by Abd El Tawab et al. [53], while Bushen et al. [54] recovered *E. coli* from broiler-dropping samples with an incidence rate of 39.0%. Differences in the isolation rate of *E. coli* may be due to early therapeutic and prophylactic use of antibiotics, the immune status of broiler chickens on the farm, hygienic measures, and management on the farm. Our serological typing study of *E. coli* isolates revealed that the examined samples belonged to O17: H18, O121: H7, O78, O127: H6, O91: H21, O103: H2, and O159. Serotype O78 was the most prevalent, with 30%, followed by O91: H21 with 20%, followed by O17: H18 with 18%. O17 was isolated and detected only in liver samples; O78 and O91 were isolated from liver and intestinal samples, while O121 and O159 were isolated from intestinal samples. A similar study by Younis et al. [55] reported the isolation ofO78 and O127 from broiler colibacillosis. Additionally, Amer et al. [56] recovered O78, O86, O158, O127, O91, O25, and O119, while Ibrahim et al. [57] detected other serotypes as O1,O2, O9, O18, O25, O26, O78, O111, O114, O119, and O127 from broiler chicken. Differences in the serotypes distribution may be due to variations in the time of sample collection and isolation, depending on the variation of isolation area.

The pathogenicity of APEC strains is related to the expression of several putative virulence genes (Table 2). Outer membrane protease (*ompA*) and increased serum survival (*iss*) are associated with protecting/serum resistance genes responsible for resistance to innate immunity and increased survival activity of bacteria in host serum. *ibeA* is functional for cell invasion into the host tissues, Hemolysin A (*hlyA*) creates pores in membranes of host cells (cell lysis), while fimbrilar *papC* (pyelonephritis associated Pili) gene stimulates the production of cytokines by T lymphocytes and colonization factor in extraintestinal infections [58]. In the present study, PCR detection and amplification of five virulence genes including (*ibeA, hlyA, iss, papC and ompA*) that represent and constitute different mechanisms of virulence in the majority of APEC strains revealed that*hlyA* gene was the most frequently expressed in all serotyped isolates (O17, O78, O91, O103, and O159) of APEC followed by *iss* and *papC* genes which were found in serotyped strains O17, O78, and O121, while *ibeA* gene was amplified and detected only in serotype O17. The *OmpA* gene is not detected in any strain serotypes. Invasions of microbes into the host tissues are essential in encouraging entry through the initial stage of infection [58]. Multiple genes encode invasions such as *ibeA* [59]. Regarding the detection of *ibeA* virulence gene markers in APEC serotyped isolates, only in serotype O17, Obata-Yasuoka et al. [60] reported that the occurrence of *ibeA* in APEC strains was 26%. Furthermore, Germon et al. [61] concluded that *ibeA* is positively associated with some strains of APEC, such as O88, O18, and O2 strains, while it was negatively associated with O78 strains. Hemolysin (*hly*) is a toxin responsible for cytotoxic activity in APEC. In our study, *hlyA* was detected with a rate of 100% in all serotypes of APEC isolates. A similar result in Egypt by Sedeek et al. [62] demonstrated that the virulence gene hemolysin *hlyF* was detected at a rate of 100% in all APEC isolates (O78a, O1, O26, and O78b), while Knopl et al. [63] reported that the occurrence of *hly in* APEC was (34%) and in another study by Johnson et al. [64] the incidence of *hlyF* in APEC was 78.2%. Another study in Egypt by Helal, [65] reported that the *iss* gene is APEC’s most significant and frequently distributed virulence marker. Moreover, Wilczynski et al. [66] showed that *the iss gene was APEC’s most frequently amplified gene*. Regarding ompA in our study, it was not detected in any isolated *E. coli*, while De Carli et al. [67] recorded 100% of *ompT* within the APEC strains. Mohamed et al. [68] recorded *iss* and *papC* in APEC isolated from broilers. Furthermore, Abd El-Tawab et al. [69] recorded *iss* and *ompA* in broiler chicken in Egypt. Detection and amplification of these virulence genes in APEC strains may differ according to the geographic region and season or year of the isolation.

Regarding serogroup O17 isolated in our study, it was isolated from liver samples of broilers with colibacillosis, and it comprised four virulence genes (*ibeA, hlyA, iss*, and *papC*). Previous results by Ananias and Yano [70] stated that systemic infection of O17 was isolated from patients with sepsis and reported that 100% of the O17 strains harbored *papC*. It was previously detected as Shiga toxin-producing *E. coli* [71] and isolated from the meat of chicken [72]. Furthermore, it is isolated from farmed poultry by Samanta et al. [73]. O17 exposed multi-drug resistance and has been responsible for more severe systemic infections in humans, including the urinary tract [74], and it was linked to nonhuman reservoirs, mostly chicken and pork [75].

In our study, AG-Cu and ZnNPs were synthesized by a green method using Arabic gum as stabilizing and capping agents. NPs can be synthesized using chemical methods such as chemical precipitation [76] or physical methods, sonochemical, solvothermal, sol-gel process, and hydrothermal decomposition [77]. All these methods need either expensive instruments or complicated techniques or have potentially hazardous effects on the environment, especially in the case of large-scale production of NPs [78]. Recently, the green synthesis of the nanoparticle has been an incipient phenomenon that effectively reduces environmental risk by eradicating the hazardous components that are harmful to human health [79]. Surface modification of NPs is necessary to control morphological characters, especially size, to optimize their efficacy in addition to controlling the state of agglomeration, which hinders the application of NPs, especially as antimicrobials [80] and keeping electrostatic interactions and steric hindrance of NPs in water system depending on the polyanionic nature of Arabic gum [81].

Here, this study uses Arabic gum as stabilizing and capping agent with a ratio of 1 and 1.5% in addition to L- ascorbic acid and sodium hydroxide as precipitating agents in the case of AG-Cu and Zn NPs synthesis, respectively. Arabic gum exudates have hydrocolloid and physical emulsification properties and a complex mixture of polysaccharides and glycoprotein, so it is considered multi-functional material with good stabilizing properties and biocompatibility with non-toxic and safe nature [82]. Our method successfully synthesized AG-Cu and Zn NPs in sizes of 58 and 75 nm, respectively, with a narrow size distribution (PDI) of 0.16 and 0.15, respectively. Surface charge of -24.8 and − 25.5 mV (Fig. 2; A1 and A2) respectively by Zetasizer, which was confirmed by TEM (Fig. 3; A1, 2 and B1, 2) that showed distinctive single well-dispersed NPs with a spherical shape, and without aggregation, and their size ranged from 7.68 to 19.5 nm for CuNPs and from 23.4 to 39.6 nm for ZnNPs. There is a direct relationship between the amount of added Arabic gum and the size of NPs resulting from aggregation in the case of increasing Arabic gum percent [83]. It was mentioned that a lower amount of Arabic gum helps in covering ZnNPs with a thin layer and allowing little particles to adhere together during the growth step when NPs bond with functional groups of Arabic gum such as carboxyl, hydroxyl, and amine groups, leading to avoiding the excess growth [83]. Moreover, 1% of Arabic gum can transform the charge dispersal of CuNPs, directly affecting the nucleation and monodispersed nature of CuNPs [27]. Nanoparticles synthesized in the presence of Arabic gum as stabilizing agents usually have negative charges on their surface resulting from the interaction of arabinogalactan proteins of AG with polysaccharide constituents that expand into the aqueous solution, giving electrostatic stability [27].

The XRD pattern of the AG-Cu and ZnNPs (Fig. 2; B1 and B2) revealed characteristic peaks (marked by red) of the metallic cubic and hexagonal phase of Cu and Zn NPs, respectively, corresponding to 2θ values of 43.3, 50.4, 74.1, 89.9, and 95.1° for CuNPs and 18.81, 32.34, 38.15, 50.86, 57.68, 61.23, 68.64, and 70.96°for ZnNPs at a wavelength of 1.54060corresponding to reference code (COD-9,012,043) for CuNPs as mentioned by Otte, [84] and (COD-1,529,590) for ZnNPs as mentioned by Baneeva and Popova, [85].

The role of Arabic gum in the synthesis of Cu and Zn NPs was identified by comparing the FT-IR spectra (Fig. 2; C1 andC2) of Arabic gum as stabilizing and capping agent with AG-Cu and Zn NPs. For Arabic gum, specific peaks of the N-H bond in stretching mode, O-H bond in stretching mode, N-H bond which bent in primary amine, and C-O bond in the stretching mode were observed at 3433, 2923, 1614, and 1070 cm^− 1^, respectively compared with 3430, 2918, 1627 and 1036 cm^− 1^ for AG-CuNPs and 3431, 2920, 1630 and 1118 cm^− 1^, respectively for AG-ZnNPs. The shifting in the peak of the N-H bond in the stretching mode of AG stabilized NPs compared with AG may be resulted from an interaction between AG protein and NPs [26]. The shifting in the peak of the C-O bond in the stretching mode of AG stabilized NPs compared with AG indicates new bond formation, and as it was reported by Geetha et al., [26], the higher shifting frequency indicates structural changes related to binding between AG and NPs. FT-IR study results confirmed that Arabic gum’s role in stabilizing Cu and Zn NPs was successfully achieved.

Different decisive factors impact the cytotoxicity of NPs, especially shape, surface charge, size, surface modification and stabilization, and time-dose dependent effect. Therefore, nanoparticle characteristics like morphology, surface charge, size, chemical structure, and agglomeration state should be considered before cytotoxicity or *in-vivo* experimentation [86]. The most commonly mentioned theoretical mechanisms in the literature are: firstly, metal ions uptake (translocation and particle internalization) into cells, followed by a decline of intracellular ATP production and interruption of DNA replication [87]; secondly, production of reactive oxygen species (ROS) from metal NPs and metal ions, with subsequent oxidative damage to cellular structures [88];and finally, accumulation and dissolving of metal NPs in the bacterial cell causing the disturbance in permeability and cellular components [89]. For CuNPs, the cytotoxicity is mainly related to oxidative stress either directly by activation of ROS generation, mainly superoxide, hydroxyl, and hydrogen peroxide free radicals, or indirectly by stimulating the cell redox system causing ROS to release, which damages cellular components, mainly DNA causing oxidative DNA damage and genotoxicity [86]. For ZnNPs, the primary mechanism of its cytotoxicity is the induction of oxidative stress [90] and free radicals generation. Our results revealed that the survival rate of OEC cell culture incubated with AG-stabilized CuNPs with different concentrations decreased from 98.7 to 81.25% at concentrations of 0.31 and 50 mg/ml, respectively. For AG-ZnNPs, the survival rate decreased from 101.82 to 89.23% at concentrations of 0.31 and 50 mg/ml (Fig. 3C). The relationship between the surface functionalization of NPs using capping agents and nano cytotoxicity is closely related. It was reported that long-chain capping agents could reduce ROS generation and inhibit cytotoxicity even at high concentrations [91]. Arabic Gum, as a long-chain polysaccharide used as a capping and stabilizing agent in the synthesis of Cu and Zn NPs, helps in reducing cytotoxicity in a high concentration of less than 50 mg/ml due to controlling free radicals production, NPs redox potential, sharing in catalytic cycle and subsequently toxic components production [92].

AST of commercial antibiotics or AG-ZnNPs indicated the complete resistance of all of the tested *E. coli* strains; O17, O78, O91, O121, and O159, to six antibiotics; amoxiclav, cefotaxime, gentamicin, doxycycline, sulphamethoxazole-trimethoprim (cotrimoxazole), and enrofloxacin from six different families besides all AG-ZnNPs concentrations (Fig. 4; A-C). However, AG-stabilized CuNPs have to ascend antimicrobial efficacy against the tested isolates at all concentrations from 20 to 100 mg/ml (Table 3), where its IZDs are considered effective as commercial regular used antimicrobials. The *E. coli* O17 strain used in our *in-vivo* study showed that the highest IZD ranged from 19.66 ± 0.33 to 25.00 ± 0.00 mm, corresponding to the used concentration. MIC and MBC of AG-Cu NPs varied according to the *E. coli* tested strains. MIC values ranged from 3.75 to 15 mg/ml, while the bactericidal effect of AG-Cu NPs (MBC) was 60 mg/ml for O17, O78, and O121; other O91 and O159 required higher concentrations than 60 mg/ml. Several concerns have been raised about the emergence of multiple antibiotic resistance (MAR) pathogens due to the heavy and random use of some of the most commonly used antimicrobials in poultry farms as the first choice for bacterial infection treatment. The responsible use of antibiotics should be adopted, and alternative antimicrobial agents with more potent immune-stimulatory properties necessary to combat *E. coli* infection should be applied besides routine monitoring of drug susceptibility patterns overtime to avoid antibiotics resistant mutants. In literature, AST of various isolated avian *E. coli* serotypes from colibacillosis cases of broilers outlined different percentages of resistance and sensitivity due to strain differences. Similarly, Ramchandani et al. [93] reported that 56% of human-associated multidrug-resistant uropathogenic *E. coli* were O17, which has an animal origin from 24 chicken samples, were resistant to trimethoprim-sulfamethoxazole, ampicillin, ciprofloxacin, amoxicillin/clavulanic acid, tetracycline, chloramphenicol, gentamicin, cephalothin, streptomycin, and kanamycin. Our antibiogram-resistant results related to some extent with Adam et al. [1], where some *E. coli* isolates were highly resistant (75%) to 9 out of 12 different tested antibiotics. Isolates revealed resistance to cephalothin, lincomycin,and erythromycin (100%), doxycycline (60%), amoxiclav(40%), and enrofloxacin, neomycin, streptomycin, and tetracycline (20%). Massot et al. [94] found that 44.8% of the collected strains showed one or more antibiotic resistances among the ten tested ones (penicillins, cephalosporins, carbapenems, aminoglycosides, tetracyclines, sulfonamides, amphenicols, quinolones, phosphonic acid, and furans). In comparison, 2.2% of all strains were multi-resistant to at least penicillins, cotrimoxazole, and quinolones.

Concerning our NPs antibiogram, Abdollahi et al. [95] recorded that antimicrobial activity IZDs of CuNPs against *E. coli* among other pathogens ranged from 20 to 32 mm. Moreover, the high *in-vitro* bactericidal efficiency of CuNPs against *E. coli* was recorded [96, 97]. Lower MIC 120 and MBC 160 µg/ml of cupric oxide NPs for *E. coli* were recorded by Chakraborty et al. [98]. For CuNPs, Su et al. [99] reported that its intracellular penetration caused a significant alteration of bacterial key protein expressions followed by a significant alteration in the metabolic growth processes. Radi et al. [100] evidenced the antibacterial activity of ZnNPs against *E. coli*. The difference in our antibacterial susceptibility between AG-Cu and Zn NPs stabilized by the Arabic gum may be attributed to their different NPs size, which could limit the entrance and the non-specific transport across the bacterial cell membrane [101].

Furthermore, Azam et al. [102] clarified that the antimicrobial activity of ZnNPs increased against gram-negative bacteria by decreasing their particle size. Hence, AG-CuNPs are more effective against *E. coli* variant strains than AG-ZnNPs. The smaller the NPs, the larger the surface area and surface reactivity, and subsequently, the high production of hydrogen peroxide free radicals due to a high number of NPs per unit volume [103], causing inhibition of *E. coli* bacterial growth. It was reported that the small size of AG-ZnNPs at 16 nm and AG-CuNPs at 12 nm have the highest antibacterial efficacy against *E. coli* bacteria due to high hydrogen peroxide free radicals generation causing bacterial damage [83].

TEM analysis of thin sections prepared from E. coli O17 cultures after treatment with 50 mg/ml AG-CuNPs (Fig. 5; A-F) revealed anchoring and accumulating NPs around the bacterial cell wall with bacterial cell deformity and elongation. Additionally, it showed intracellular distribution in the cytoplasm with a ghostly appearance devoid of cytoplasmic content, a destabilization of the cellular membrane resulting in low electronic density, a faint appearance of cytoplasm, and cell damage. These findings are similar to those explained by Díaz-Visurraga et al. [104], who showed that the first interaction of metal NPs with the cell wall results in exopolysaccharide matrix fragmentation, cell separation, elongation, and cell reorganization into small clusters. These modifications enhance the surfaces of physically interacting bacterial cells with nanostructured materials. Ghost formation seems to be another morphological feature related to the NPs toxicity in bacterial cells. Indeed, a cell ghost is an intact surface structure lacking cytoplasmic content, including DNA mainly from gram-negative bacteria, but not always. The deformity of the cell’s physical structure may cause the membrane to swell and disturb, increasing membrane fluidity, which in turn raises passive permeability and demonstrates as leakage of various critical intracellular constituents like ATP, DNA, sugar content, enzymes, and amino acids [104].

Antibacterial performance assay of AG-CuNPs on *E. coli* O17 (Fig. 6; A and B) displayed the effect of different (*E. coli*-AG-CuNPs) contact time and AG-CuNPs concentrations on the viability of *E. coli* O17 to meet the bactericidal activity threshold. It was found to be increased with prolonged time and higher concentrations. The antibacterial rate reached 100%, at 20 min and 40 mg/ml AG-CuNPs. Other literature reported that the antibacterial effect of CuNPs against *E. coli* is complementary action dependent on the concentration of NPs. A higher concentration will be more effective, where a large surface area to volume ratio helps in bacterial cell wall damage and cell death [105]. Besides the sudden decline in membrane integrity, the release of ROS contributes to the degradation of several biomolecules that affect normal cell viability [106]. Compared to other studies, our time-kill and concentration results varied from that of Li et al. [33], who reported that antibacterial efficiency reached nearly 100% after 28 min at 4.0 g/L CuNPs, while Du et al. [107] recorded it after 4 h exposure at 500 mg/l and40 nm. These discrepancies are possibly attributable to the variation in NPs size, *E. coli* strain, and the synthetic method used.

*E. coli* O17 serotype has been used for experimental broiler challenge and to evaluate the effect of AG-CuNPs on these virulence genes because it is the highest strain in the number of detected virulence genes (4 genes). In this study, the qRT-PCR was used to investigate the frequency and fold change of the virulence genes in the APEC O17 strain before and after treatment with AG-CuNPs. Our study explored that virulence genes (*ibeA, hlyA, iss*, and *papC*) were significantly down regulated in the treated *E. coli* strain sample compared to the control non-treated strain after the treatment dose of AG-CuNPs (50 mg/ml) (Fig. 7). A similar result was reported; CuNPs, compared with silver NPs, were more effective on the *E. coli* genome [108]. Another study by Awaad et al. [109] demonstrated that silver NPs down regulated *iss, papC, iutA, iron*, and *fimH* virulence genes expression.

Our study recorded negative impacts post virulent *E. coli* O17 infection, such as severe retardation in growth, feed intake, organs’ lesion score, and mortalities; similar findings were reported by Rosa et al. [110]. On the other hand, treated group G4 had no mortality with a general improvement in broilers’ activity that may be attributed mainly to the curative antibacterial activity of the used NPs on pathogenic *E. coli* or its virulence genes. As well, El-kazaz and Hafez [111] observed the positive effect of CuNPs on all broilers’ behavioral patterns (feeding, drinking, crouching, resting, and movement) post their addition to drinking water (10 mg/l) for five weeks, attributing these results to the significant reduction of serum corticosterone values which might indicate an inhibition of the stress condition. Moreover, Sarvestany et al. [112] found that even at a dose of 100 mg/kg, CuNPs had no adverse effects when used as an alternative to antibiotics in poultry.

Our results displayed that *E. coli* re-isolation and count were markedly decreased in infected treated G4 compared to infected control G2 in its intestinal count. At the same time, no *E. coli* was re-isolated from liver samples, which assured the efficacy of AG-CuNPs in stopping the systemic infection of *E. coli* O17 *in-vivo*. Our *in-vivo* findings are long-established with our antibacterial activity regarding *in-vitro* tests and TEM analysis. Similarly, Hassanen et al. [7] found that oral doses of CuNPs 5 mg/kg body weight for seven days reduced 50% of *E. coli* O78 count in blood, liver, and other organs than challenged untreated broilers. Hence, CuNPs could penetrate the intestinal walls, where smaller constituent parts can diffuse faster through the GIT mucus to directly enter the bloodstream, after that being pre-dominantly scavenged by the liver and spleen [113].

Our results showed a significant decrease in TP, albumin, globulin, and lysozyme activity besides an increase in C-reactive protein, ALT, GGT, and ALP activities, uric acid, and creatinine in infected control G2 than control G1 (Tables 4 and 5). Post-treatment, non-infected treated G3 revealed no significant difference than G1 in TP, albumin, globulin, uric acid, or creatinine, while significantly decreased C-reactive protein levels and increased lysozyme activity and most of the activities of the detected enzymes; at the end of the experiment results mostly returned to its normal levels. Infected treated G4 showed a significant improvement in most of the detected parameters than G2 by increasing TP, albumin, globulin, and lysozyme activity besides decreasing C-reactive protein, uric acid, creatinine, and most of the activities of the detected enzyme than G2. In contrast, it showed no significant difference from G1 in most of these detected parameters at the end of the experiment. Concerning changes in liver biomarkers, proteins are considered the terminal executors of specific biological processes, such as catalyzing metabolic reactions and transporting substances [99], while ALT and GGT values are considered two proper hepatic function evaluation tests; hence, an increase in their activities is indicative to hepatocellular injury [114].

Moreover, Balakrishna and Prabhune [115] mentioned that GGTs are highly conserved enzymes that play an essential role in glutathione homeostasis (a major cellular antioxidant) due to some of the common physiological γ-glutamyl substrates like glutathione. Regarding G2, the detected virulence genes of *E. coli* O17 confirmed its systemic *in-vivo* consequences on vital organs. Manafi et al. [116] reported a similar increase in ALT, and ALP, attributing these influences to *E. coli* endotoxins that passed through the intestinal wall to the blood, causing consequent damage to liver and kidney organs, which appeared as severe to moderate pathological alterations as found by Hassanen et al. [7].

Our findings of treated groups G3 and G4 indicated an immune stimulant effect of AG-CuNPs without enduring adverse effects on liver or kidney functions. These *in-vivo* findings coincided with our previous cytotoxicity assay. Hence, AG-CuNPs suppress the progressive destructive effects of *E. coli* through their effective antibacterial activity on the bacteria or its virulence genes. Compared with other studies, El-Kassas et al. [117] recorded that CuNPs reduce liver enzyme activities, protect the hepatic tissue from degenerative changes, and increase stress resistance. Moreover, Sizova et al. [103] found that CuNPs tended to increase serum TP and aminotransferase activities without changes in GGT, lactate dehydrogenase (LDH) activities, or liver microstructure of treated groups. The authors considered GGT as a marker of amino acid balance and assumed that the increase in ALT activity indicates changes in the metabolic flows of the NPs-treated groups. Hence, the effect on enzyme activity depends on NPs absorption and metabolism according to their physicochemical properties, such as size, shape, surface chemistry and charge, length, method of administration, and dose [118].

Consequently, the benefits or drawbacks of NPs on vital organs, redox reactions, and immune defense depend mainly on selecting their effective, safe dose. For example, Abdelazeim et al. [119] and Elkhateeb et al. [120] reported that high continuous daily doses of CuNPs (100 and 250 mg/kg body weight, respectively) are required to induce toxicity of the liver or kidney. On the other hand, Cholewińska et al. [121] reported that low doses of dietary CuNPs cause a significant increase in plasma AST, ALT, and ALP activities, while they decrease ALT activities at high doses. Conversely, Morsy et al. [106] observed that 15 mg/ml CuNPs revealed a remarkable improvement in the treated *E. coli-*infected broilers; it caused some pathological alterations in different organs with high Cu levels in the liver and kidneys. Other studies revealed that CuNPs reduced the level of albumin in chickens [122]; meanwhile, Scott et al. [123] found a significant decrease in ALT, AST, and uric acid levels by 20 mg/kg CuNPs and a non-significant effect on albumin, ALP, and LDH.

The present study demonstrated a significant increase in lysozyme activity of both treated groups G3 and G4 than G1 and G2 (Table 5). Elevation of serum lysozyme is a good indicator of immune system activation besides its antimicrobial mechanism. It is released from mononuclear phagocytes, which are involved in disease resistance, especially during infection. Furthermore, Lysosomes play an essential role in degrading dysfunctional proteins and damaged organelles to maintain cellular homeostasis. Besides, they are mainly involved in receiving and degrading phagocytized macromolecules [124]. Similar results were reported by El-Kassas et al. [117] besides the CuNPs’ effect on upregulating immune-modulatory genes. Besides, Arancibia et al. [125] observed that CuNPs recruit different innate immune cells, such as macrophages and neutrophils, which are crucial to initiating the immune response to different foreign molecules or harmful agents.

Moreover, the redox-active properties of MONPs tend to modulate the innate and adaptive immunity that successfully enhances the immune response [126]. The perspective of CuNPs as an immune-modulator is promising for their future use in poultry feed and medicine [13]. In contrast, lysozyme activity post-CuNPs supplementation was non-significantly changed in the previous study by El-kazaz and Hafez [111].

C-reactive protein (CRP) is γ-globulin produced by the liver and classified as an acute phase reactant, which means that it rises in response to general inflammation, infection, or tissue injury following IL-6 secretion by macrophages. It is produced in various bacterial diseases because it acts as an opsonin that helps in complement activation and has a receptor on phagocytic cells besides binding to the bacterial lysophosphatidyl choline [127]. Therefore, enhancing pro-inflammatory cytokines (CRP, IL-6, and ceruloplasmin) is vital for restoring homeostasis to normal physiological ranges [128]. However, in the inflammatory phase, ROS can damage the proteins and DNAs. Hence, the anti-inflammatory properties of AG-CuNPs (Table 5) that significantly decreased CRP levels positively impact counteracting infection subsequences, supporting vital organ functions and general health aspects besides its antibacterial activity. Similar anti-inflammatory properties and inhibition of other inflammatory markers and tissue damage by CuNPs were reported by El-kazaz and Hafez [111], El-Kassas et al. [117], and Faisal et al. [129].

Regarding the kidney biomarkers (Table 5), increased serum creatinine and blood urea nitrogen concentrations impaired renal function and thereby decreased glomerular filtration rate due to creatinine being freely filtered in kidney glomeruli and typically no tubular re-absorption [130]. Scott et al. [123] revealed that the participation of CuNPs leads to decreasing blood urea, indicating its potential association with higher protein metabolism/formation and more efficient utilization of amino acids for growth. In general, the varied and sometimes contradictory results for the effect of CuNPs on biochemical markers can be explained by variations in routes of administration, treatment dose, and duration, besides differences in NPs physicochemical properties that induced different biological reactions.

Results of Cu residues (Table 6) indicated that *E. coli* infection significantly increased Cu residues in G2 than G1 and in infected treated G4 than in non-infected treated G3. Although, in general, Cu residues in the liver were more than in muscle, all Cu levels decreased by time at the study end with no significant difference than G1 in muscles, while it needs more time to decrease in the liver organ. The absence of metal residues at the end of the study assured that small-size NPs from the lymphatic and circulatory systems might distribute to organs, including the kidneys, from where they can be rapidly cleared [113], which was assessed in our AG-CuNPs characters.

Furthermore, Cholewińska et al. [121] mentioned that hepatic Kupffer cells are of paramount importance in NPs elimination; tiny ones are filtered out of the liver through the kidneys, but bigger ones are retained in the Kupffer cells. Similarly, Sizova et al. [103] and Morsy et al. [106] detected the highest Cu content in liver tissue and the lowest in muscle. The high hepatic accumulation of MONPs may be due to the high production rate of metallothioneins (MTs) which ultimately bind the metals to avoid possible harm [131]. Moreover, the liver is a specialized organ for metal detoxification and Cu metabolism through metal sulfur-protein formation. Meanwhile, the less concentration of MONPs in the muscle may be due to the low level of metal-binding protein as MTs in the muscle and owing to the large mass with the low metabolic activity of muscular tissues [132]. The biocompatibility and toxicity of MONPs are essential criteria to take into account for their biomedical applications. Their biocompatibility is linked to the biodegradation of metabolites and the immune system response post-administration. Their toxicity profile may be increased or decreased due to modifying their cell/tissue biodistribution and clearance/metabolization. Hence, the size and distribution, besides shape, are the crucial objects influencing the pharmacokinetics and biodistribution *in-vivo* applications and determining their fate.

Additionally, the surface charge plays a vital role in the physical stability and influences its interaction with the biological system and its safety. Similarly, Sawosz et al. [133] and El-Kassas et al. [117] found no change in Cu levels of breast muscle or liver to control post-feeding with CuNPs at 7.5 mg/kg or 100% of the daily requirement for 42 days, respectively. Moreover, Ognik et al. [134] showed that shortening the period of CuNPs administration to seven days decreased its accumulation. Furthermore, Ognik et al. [135] reported that once the level of Cu is too high, its absorption and accumulation probably decrease while its excretion increases to ensure normal metabolism. Besides, an additional increase of CuNPs than the recommended NRC amount showed no linear increase in the content of Cu in the liver or breast muscle [135]. On the contrary, Hassanen et al. [7] found a significant increase in Cu content in muscles with no significant difference in the liver. In addition, Sizova et al. [136] mentioned that CuNPs have high penetration ability through the intestines, bypassing traditional binding and transport by proteins, consequently giving a more consistent supply of Cu.

## Conclusion

Multi-resistant *E. coli* strains have emerged as a serious worldwide problem. Most of the available antibiotics have lost their potency over *E. coli* due to their abuse, which could be found clearly in antibiogram tests. Hence, there is a growing trend to replace ineffective antimicrobials with alternative materials to intervene in a strategy for controlling bacterial infection in poultry farms. This study was designed to compare the efficiency of AG-Zn, and Cu NPs against various field-isolated APEC strains. AG-CuNPs were prepared through a green, eco-friendly, and safe method. From our results, we concluded that the morphology of AG-CuNPs showed smaller particle size and homogeneous distribution than AG-ZnNPs which signifies the antibacterial efficacy of Cu than Zn NPs against tested strains (O17, O78, O91, O121, and O159). Furthermore, the AG-CuNPs exhibited low cytotoxicity, efficient bactericidal activity at TEM, and antibacterial performance assays besides their anti-inflammatory and immune-modulator properties.

Moreover, it successfully decreased the expression of detected (*ibeA, hlyA, iss*, and *papC*) virulence genes of the *E. coli* O17 strain. Additionally, it neither affects liver or kidney functions nor leaves residues in muscles. It restored most of the tested biomarker levels to near the normal control values. Therefore, we highly recommend using AG-CuNPs as an alternative antibacterial agent (nanobiotic) in treating poultry-associated *E. coli* pathogens. AG-CuNPs have broad potential application prospects. Consequently, more studies are needed to compare their *in-vitro* and *in-vivo* effects against various economically critical bacterial infections of poultry production.

### Electronic supplementary material

Below is the link to the electronic supplementary material.

**Additional file 1: Supplementary Fig. 1**: A schematic illustration of the experimental design.

**Additional file 2: Supplementary Fig. 2**: Agarose gel electrophoresis for PCR amplification of hlyA virulence gene at (1177 bp) in APEC serotypes: Lane L (ladder), Lane + C (control positive), Lane - C (control negative), Lane 1 (serotype O17), Lane 2 (serotype O78), Lane 3 (serotype O91), Lane 4 serotype (O121) and Lane 5 (serotype O159). **Supplementary Fig. 3**: Agarose gel electrophoresis is for PCR amplification of ***ibeA*** virulence gene at **(342 bp)** in APEC serotypes: Lane L (ladder), Lane + C (control positive), Lane - C (control negative), Lane 1 (serotype O17), Lane 2 (serotype O78), Lane 3 (serotype O91), Lane 4 serotype (O121) and Lane 5 (serotype O159). **Supplementary Fig. 4**: Agarose gel electrophoresis for PCR amplification of ***iss*** virulence gene at **(309 bp)** in APEC serotypes: Lane L (ladder), Lane + C (control positive), Lane - C (control negative), Lane 1 (serotype O17), Lane 2 (serotype O78), Lane 3 (serotype O91), Lane 4 serotype (O121) and Lane 5 (serotype O159). **Supplementary Fig. 5**: Agarose gel electrophoresis for PCR amplification of ***papC*** virulence gene at **(200 bp)** in APEC serotypes: Lane L (ladder), Lane + C (control positive), Lane - C (control negative), Lane 1 (serotype O17), Lane 2 (serotype O78), Lane 3 (serotype O91), Lane 4 serotype (O121) and Lane 5 (serotype O159).

## Data Availability

The corresponding author will provide the datasets used in this study on reasonable request.
