# Supplementary figures and images for: Efficacy of zinc oxide and copper oxide nanoparticles on virulence genes of avian pathogenic E. coli (APEC) in broilers
Source: BMC Vet Res. 2023 Aug 4;19:108. doi: 10.1186/s12917-023-03643-y (PMC10401765; doi:10.1186/s12917-023-03643-y)

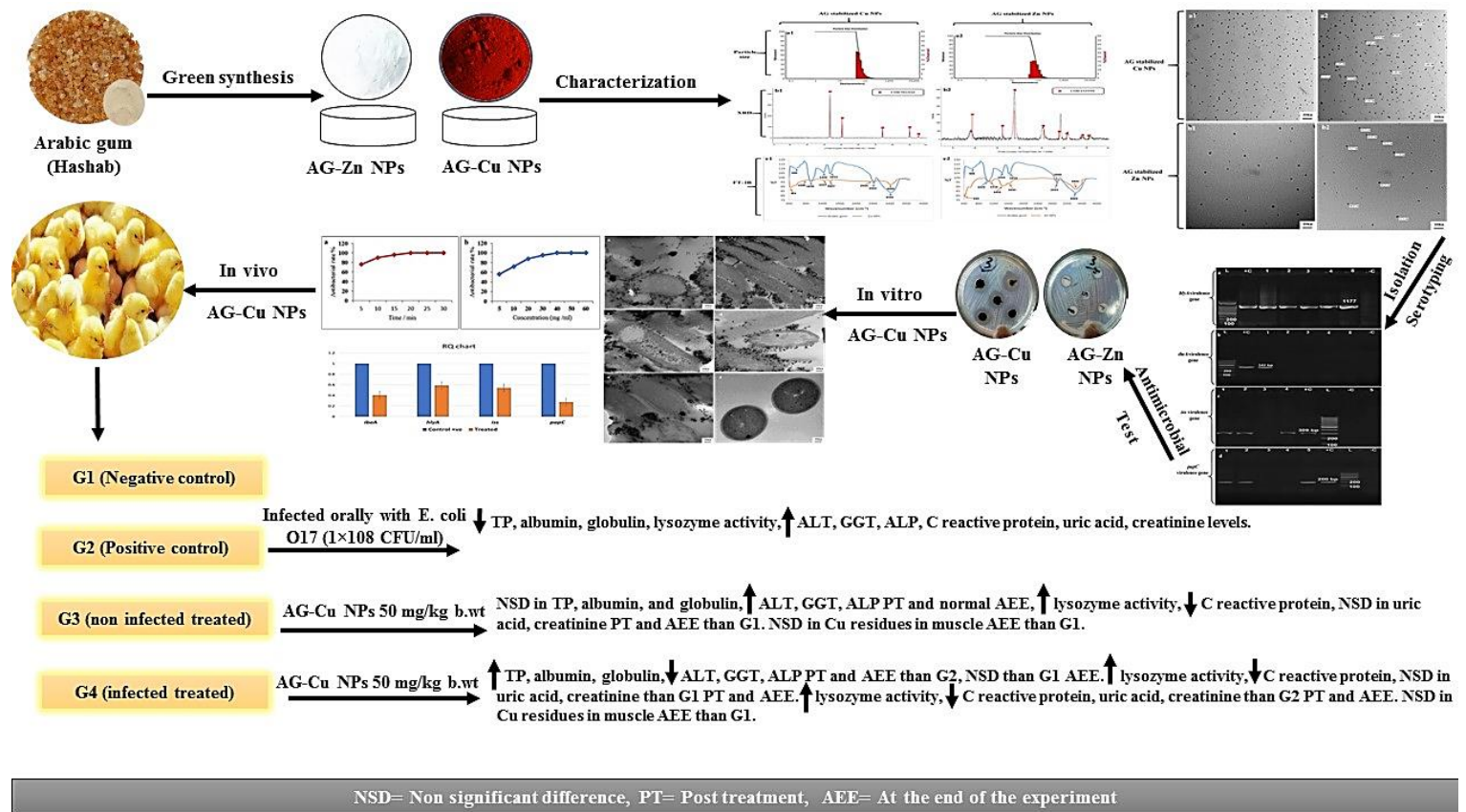

**Supplementary Figure 1:** A schematic illustration of the experimental design.

Supplement: Supplementary file 1 — Additional file 1: Supplementary Fig. 1: A schematic illustration of the experimental design. [file 12917_2023_3643_MOESM1_ESM.pdf]
