# Supplementary material for: Efficacy of zinc oxide and copper oxide nanoparticles on virulence genes of avian pathogenic E. coli (APEC) in broilers
Source: BMC Vet Res. 2023 Aug 4;19:108. doi: 10.1186/s12917-023-03643-y (PMC10401765; doi:10.1186/s12917-023-03643-y)
Supplement: Supplementary file 2 — Additional file 2: Supplementary Fig. 2: Agarose gel electrophoresis for PCR amplification of hlyA virulence gene at (1177 bp) in APEC serotypes: Lane L (ladder), Lane + C (control positive), Lane - C (control negative), Lane 1 (serotype O17), Lane 2 (serotype O78), Lane 3 (serotype O91), Lane 4 serotype (O121) and Lane 5 (serotype O159). Supplementary Fig. 3: Agarose gel electrophoresis is for PCR amplification of ibeA virulence gene at (342 bp) in APEC serotypes: Lane L (ladder), Lane + C (control positive), Lane - C (control negative), Lane 1 (serotype O17), Lane 2 (serotype O78), Lane 3 (serotype O91), Lane 4 serotype (O121) and Lane 5 (serotype O159). Supplementary Fig. 4: Agarose gel electrophoresis for PCR amplification of iss virulence gene at (309 bp) in APEC serotypes: Lane L (ladder), Lane + C (control positive), Lane - C (control negative), Lane 1 (serotype O17), Lane 2 (serotype O78), Lane 3 (serotype O91), Lane 4 serotype (O121) and Lane 5 (serotype O159). Supplementary Fig. 5: Agarose gel electrophoresis for PCR amplification of papC virulence gene at (200 bp) in APEC serotypes: Lane L (ladder), Lane + C (control positive), Lane - C (control negative), Lane 1 (serotype O17), Lane 2 (serotype O78), Lane 3 (serotype O91), Lane 4 serotype (O121) and Lane 5 (serotype O159). [file 12917_2023_3643_MOESM2_ESM.pdf]

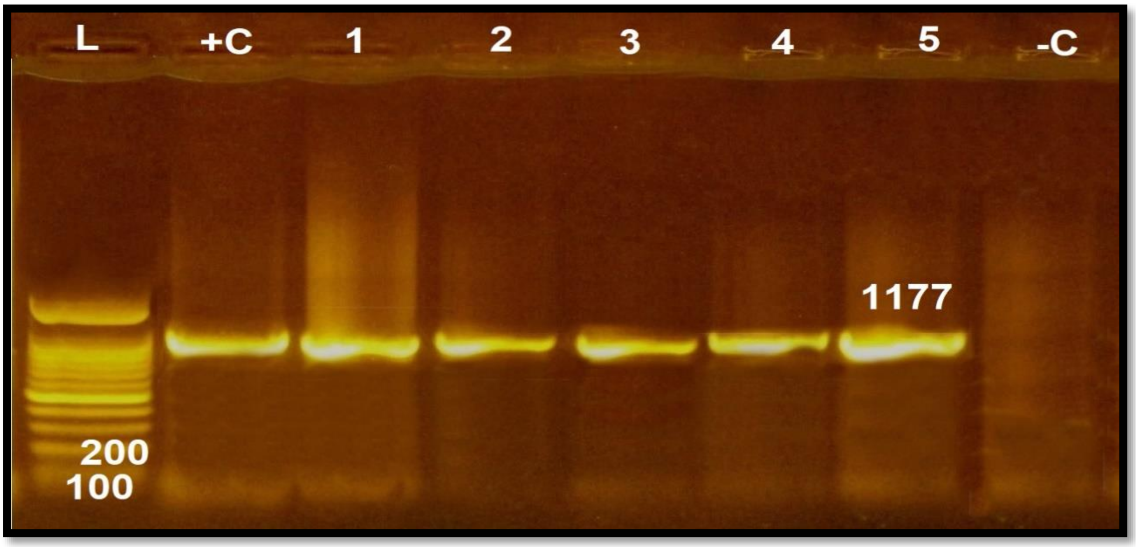

Supplementary Figure 2: Agarose gel electrophoresis for PCR amplification of ***hlyA*** virulence gene at **(1177 bp)** in APEC serotypes: Lane L (ladder), Lane +C (control positive), Lane - C (control negative), Lane 1 (serotype O17), Lane 2 (serotype O78), Lane 3 (serotype O91), Lane 4 serotype (O121) and Lane 5 (serotype O159).

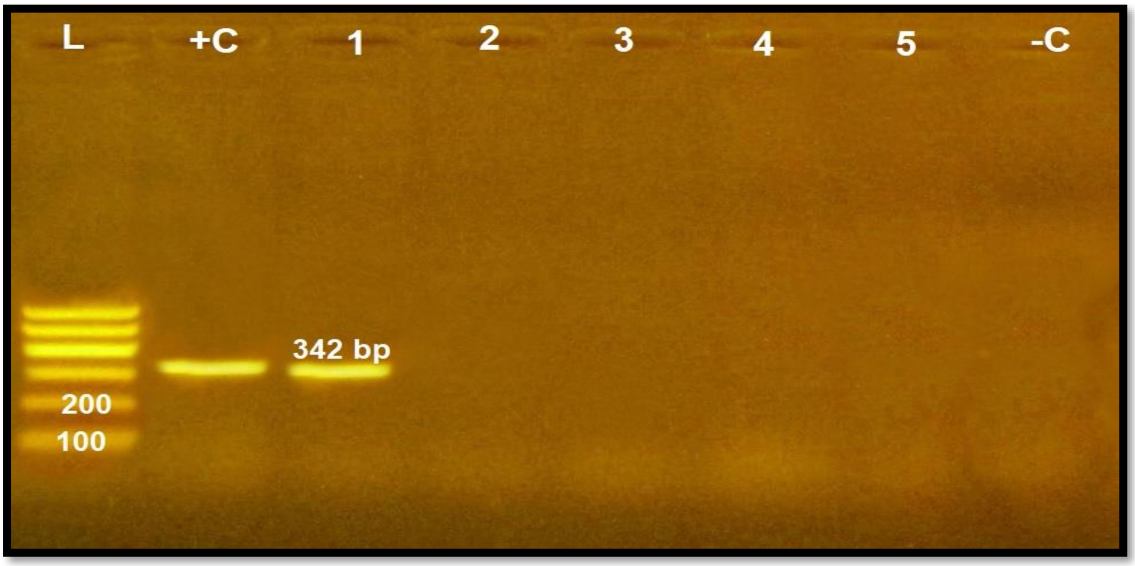

Supplementary Figure 3: Agarose gel electrophoresis is for PCR amplification of ***ibeA*** virulence gene at **(342bp)** in APEC serotypes: Lane L (ladder), Lane +C (control positive), Lane - C (control negative), Lane 1 (serotype O17), Lane 2 (serotype O78), Lane 3 (serotype O91), Lane 4 serotype (O121) and Lane 5 (serotype O159).

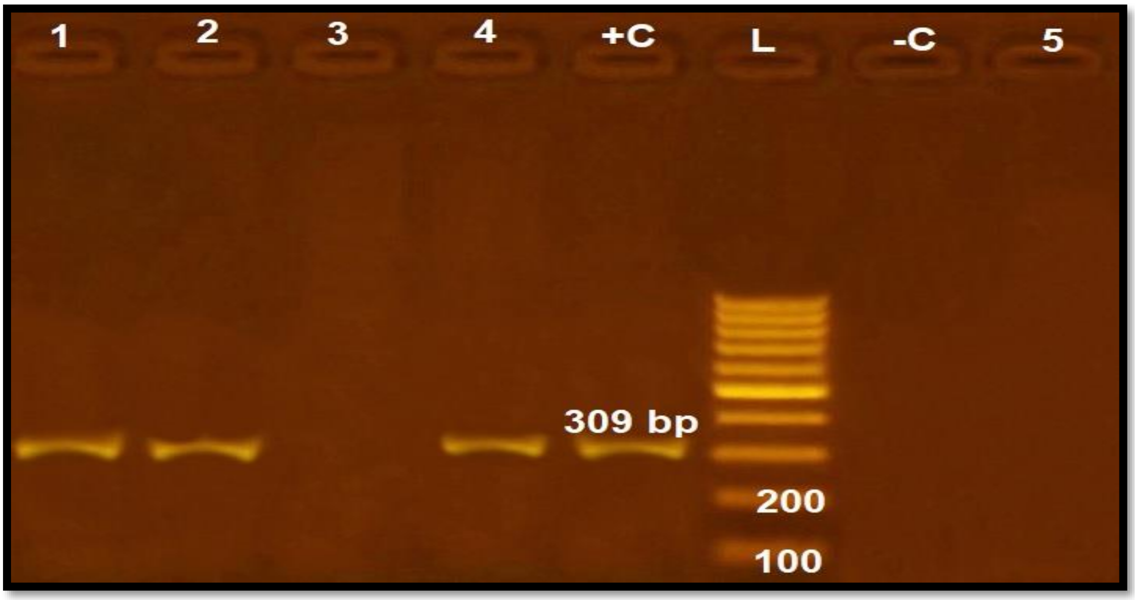

Supplementary Figure 4: Agarose gel electrophoresis for PCR amplification of ***iss*** virulence gene at **(309 bp)** in APEC serotypes: Lane L (ladder), Lane +C (control positive), Lane - C (control negative), Lane 1 (serotype O17), Lane 2 (serotype O78), Lane 3 (serotype O91), Lane 4 serotype (O121) and Lane 5 (serotype O159).

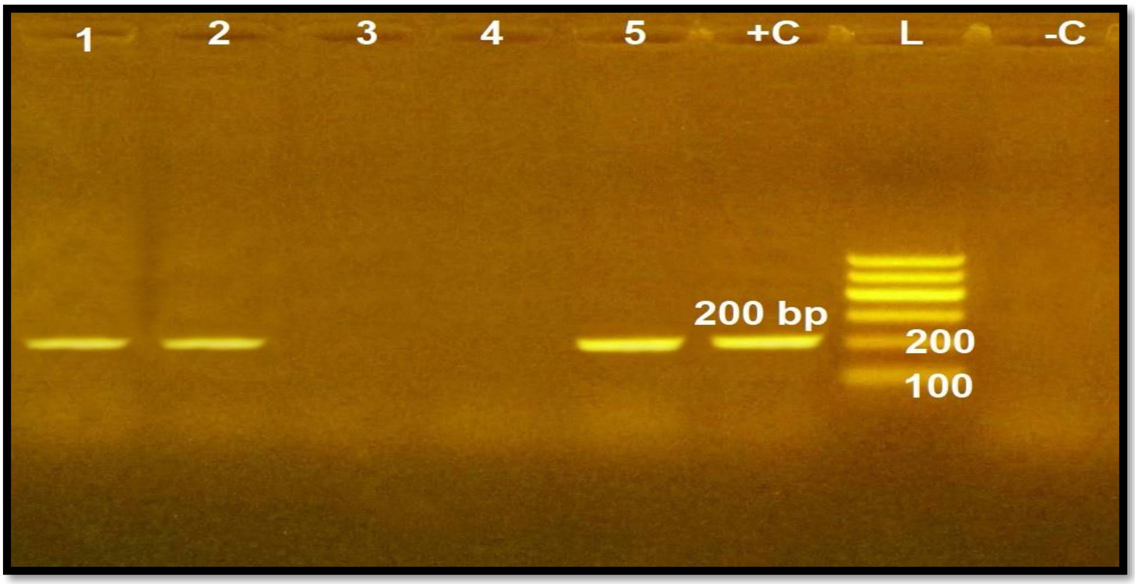

Supplementary Figure 5: Agarose gel electrophoresis for PCR amplification of ***papC*** virulence gene at **(200 bp)** in APEC serotypes: Lane L (ladder), Lane +C (control positive), Lane - C (control negative), Lane 1 (serotype O17), Lane 2 (serotype O78), Lane 3 (serotype O91), Lane 4 serotype (O121) and Lane 5 (serotype O159).
